# Palladium(II)‐Catalysed Aminocarbonylation of Terminal Alkynes for the Synthesis of 2‐Ynamides: Addressing the Challenges of Solvents and Gas Mixtures

**DOI:** 10.1002/cssc.201601601

**Published:** 2017-01-23

**Authors:** N. Louise Hughes, Clare L. Brown, Andrew A. Irwin, Qun Cao, Mark J. Muldoon

**Affiliations:** ^1^ School of Chemistry and Chemical EngineeringQueen's University of BelfastStranmillis RoadBelfast, David Keir BuildingBT9 5AGNorthern Ireland

**Keywords:** alkynes, carbonylation, oxidation, palladium, solvents

## Abstract

2‐Ynamides can be synthesised through Pd^II^ catalysed oxidative carbonylation, utilising low catalyst loadings. A variety of alkynes and amines can be used to afford 2‐ynamides in high yields, whilst overcoming the drawbacks associated with previous oxidative methods, which rely on dangerous solvents and gas mixtures. The use of [NBu_4_]I allows the utilisation of the industrially recommended solvent ethyl acetate. O_2_ can be used as the terminal oxidant, and the catalyst can operate under safer conditions with low O_2_ concentrations.

2‐Ynamides are valuable building blocks in the synthesis of heterocycles^1^ and biologically active molecules.^2^ There are a number of methods for the preparation of 2‐ynamides. Examples of non‐catalytic approaches include the coupling of alkynyl carboxylic acids with amines, using *N*‐hydroxysuccinimide and the coupling reagent *N*,*N*′‐dicyclohexylcarbodiimide (DCC) in 1,4‐dioxane.1b Terminal alkynes, amines and CO were utilised to prepare 2‐ynamides by Hoberg and Riegel; however, they employed Ni^II^ complexes as a stoichiometric reagent.^3^ Ideally, 2‐ynamides should be prepared using catalytic methods, and there are a variety of examples that were reported. Pd/Cu catalyst systems were used for the reaction of carbamoyl chlorides with terminal alkynes;^4^ however, the use of such acid chloride derivatives is undesirable. There are a number of more recent reports exploring catalytic alternatives. Dong et al. demonstrated a catalytic system comprised of bromoalkynes and amines utilising Pd_2_(dba)_3_, Xphos and Cs_2_CO_3_ as the catalyst and Co_2_(CO)_8_ as the carbonyl source (Scheme 1 A).^5^ Lee and co‐workers reported the use of alkynyl carboxylic acids and amines with CO gas, using Ag_2_O as a base and oxidant (Scheme 1 B).^6^ Ye and co‐workers used a Cu catalyst for the cross dehydrogenative coupling of terminal alkynes with formamides (Scheme 1 C).^7^ In this case a large excess of the formamide reagent was required.

**Scheme 1:**
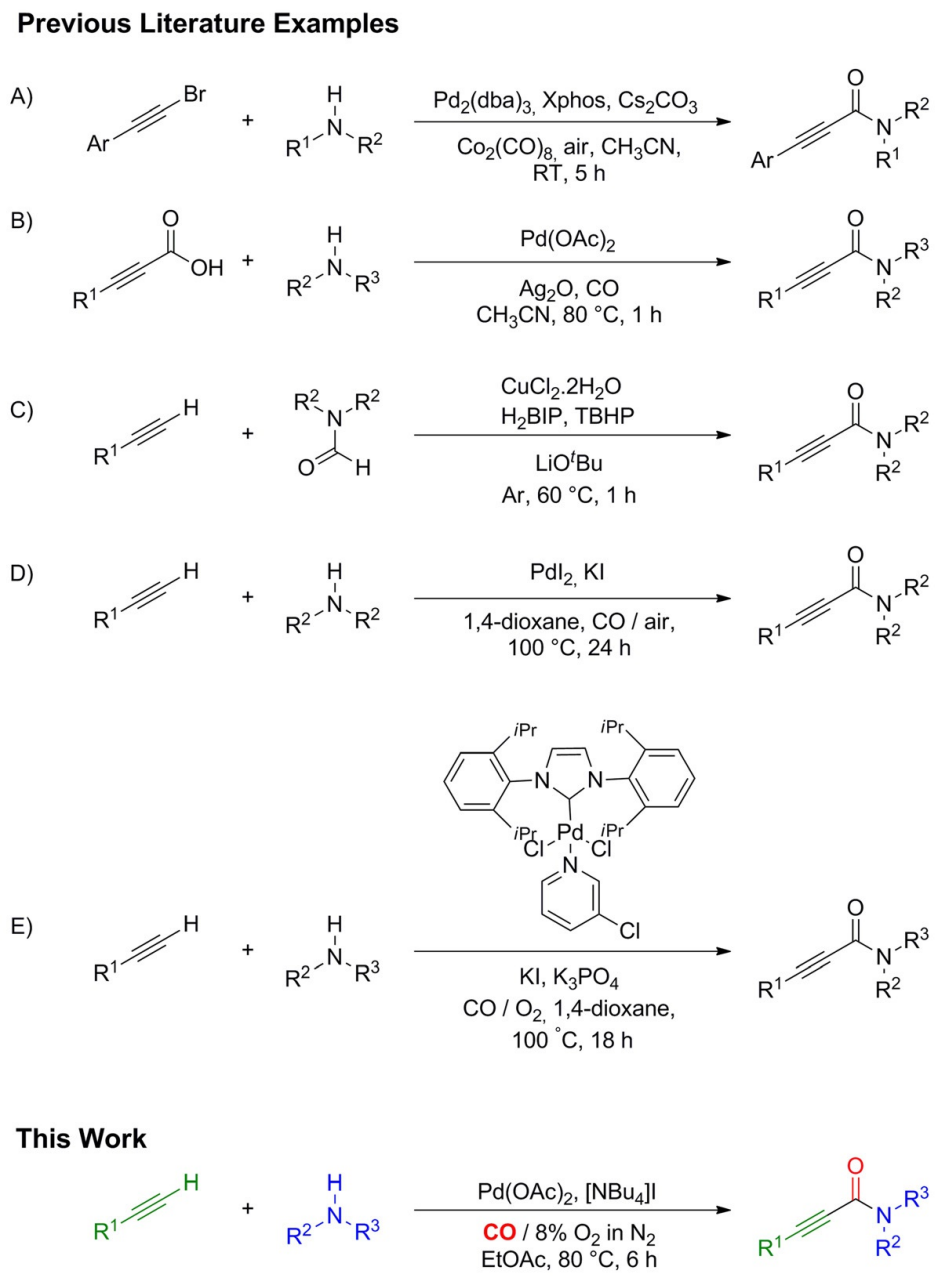
Examples of the synthesis of 2‐ynamides.

Owing to our previous work in Pd^II^ catalytic oxidations,^8^ we were particularly interested in the oxidative carbonylation methods, which use Pd catalysts with O_2_ as the oxidant and CO gas as the carbonyl source. In general, Pd‐catalysed oxidative carbonylation reactions offer potentially advantageous synthetic methods,^9^ but such reactions have been less well developed compared to other areas of Pd^II^ oxidation chemistry, such as alcohol oxidation.^10^

We recently developed a system for the synthesis of 2‐alkynoates through oxidative carbonylation of alkynes and alcohols.^11^ Moving on from this work, the aim of this study was to improve the synthesis of 2‐ynamides. There are a number of previous reports that use alkynes, secondary amines and CO to produce 2‐ynamides. This route is desirable because alkynes and amines are commercially available and inexpensive, and these reagents are used without additional functionalisation to activate them. Additionally, CO is an abundant and inexpensive source of the carbonyl group. In 2001, Gabriele et al. reported a method which utilised a simple PdI_2_‐catalyst system (Scheme 1 D).^12^ In a study focused on the preparation of 2‐alkynoates (in which the nucleophile is an alcohol), Yamamoto and co‐workers showed that their catalyst system (PdCl_2_ and triphenylphosphine in DMF) could also be used for the synthesis of 2‐ynamides, using diethylamine.^13^ Recently, Xia and co‐workers reported a Pd^II^ system with an *N*‐heterocyclic carbene ligand (Scheme 1 E).^14^ Bhanage and co‐workers reported a heterogeneous system that used Pd on carbon in 1,4‐dioxane with tetrabutylammonium iodide, [NBu_4_]I, as an additive.^15^ Recently, the same group reported the use of Pd on carbon with KI as an additive in acetonitrile.^16^ In this report they utilised tertiary amines (i.e., the reaction involved an *N*‐dealkylation step), resulting in the formation of a mixture of products in the case of unsymmetrical amines.

We felt that the work to‐date had aspects that needed to be addressed because the reaction conditions would significantly hamper the wider application of these catalytic methods. In particular, the reliance on hazardous solvents is an issue. DMF was used by Yamamoto and co‐workers,^13^ whereas the three papers which focussed on 2‐ynamide synthesis using secondary amines all employed 1,4‐dioxane. Solvents are crucial to the sustainability and safety of chemical reactions, and so it is important when optimising a catalytic method to critically evaluate the solvent selected. Solvent‐selection guides, based on safety, health and environment criteria, produced by some of the world's largest pharmaceutical companies, classed DMF and 1,4‐dioxane as “hazardous” and “problematic” and stated that such solvents should be avoided.^17^ Furthermore, ethereal solvents such as 1,4‐dioxane are arguably completely incompatible for performing oxidation reactions in a safe and scalable manner. It is well known that such solvents readily form peroxide species and laboratories have to take precautions if using such solvents in general, for example, only storing them for short periods and testing for the presence of peroxides.^18^ 1,4‐Dioxane is often reported in the academic literature as a solvent or co‐solvent in oxidation reactions, and it could be that the presence of peroxides is a factor in aiding the reactivity. There are a number of studies on Pd^II^‐catalysed oxidation systems that have investigated the role of peroxides resulting from 1,4‐dioxane.^19^ Although such systems may be academically interesting, we believe that the use of ethereal solvents for aerobic oxidation reactions is not something that would be adopted by industry. We believe that the use of such an oxygen sensitive solvent hinders this area of catalysis. In this study we have employed ethyl acetate, which is classed as a “recommended” solvent in the solvent‐selection guides developed by the pharmaceutical industry.^17^

There are additional safety issues for aerobic reactions, and the safe use of O_2_ has not been discussed in these previous oxidative carbonylation studies. Although there is no doubt that O_2_ is the most economical and sustainable terminal oxidant it does pose safety hazards, particularly if used on a larger scale. The flammability of organic solvents is one important consideration, and employing limiting oxygen concentrations (LOC) is one way to try and use O_2_ safely. The LOC of ethyl acetate at 100 °C is 9.4 vol % O_2_ at a pressure of 1 bar absolute and 9.9 vol % O_2_ at a pressure of 20 bar absolute.^20^ An additional issue with carbonylation reactions is that CO is a flammable gas. The lower flammability limit [LFL, also referred to as the lower explosion limit (LEL)] of carbon monoxide in air is 11.5 vol % at 100 °C and atmospheric pressure.^21^ The upper flamability limit [UFL, or upper explosion limit (UEL)] is 75 vol % at 100 °C and atmospheric pressure. In the case of the method by Gabriele et al. (Scheme 1 D), they utilised a gas mixture of CO and air in a 4:1 ratio at a total pressure of 20 atm.^12^ It would appear that this ratio is in‐line with the UFL (i.e., the fuel rich region). However, industry would prefer to operate within LFL conditions (fuel lean region). First of all, it is known that with increasing pressure the LFL values decrease somewhat but UFL values increase and do so to a greater extent.^22^ Additionally, if UFL conditions are used there is an inherent danger because if the vapour phase is vented (or if there is a leak) the gas mixture will pass through the flammable/explosive region as it mixes with the air. If LFL conditions are used the gas will not form ignitable compositions upon mixing with air.^23^ In this study we used 5 bar CO and 30 bar of an O_2_/N_2_ (8:92) gas mixture. As recently highlighted by Stahl and co‐workers,^20^ there is a lack of safety data under the types of reaction conditions that are used in aerobic catalytic reactions; therefore, the conditions we used have not been studied exactly. However, we based our conditions on the aforementioned LOC and LFL data, which should mean the system is safe. Importantly, it also demonstrates that the catalyst can operate effectively under such conditions.

We commenced the reaction optimisation using phenylacetylene and diethylamine as the model reaction in the presence of PdI_2_ (0.2 mol %) and [NBu_4_]I (2.5 mol %). The reaction was performed at 80 °C in EtOAc for 6 h with a 1:2 ratio of alkyne/amine.

We initially screened both 8 % O_2_ in N_2_ and air (30 bar) with CO (5 bar), finding that both afforded almost identical results. This indicated that the system was not limited in O_2_ when operating under these more dilute conditions. This is important because if the system is limited in O_2_ Pd^0^ aggregation and catalyst deactivation will occur. Moving on from this, we screened a variety of Pd salts, as shown in Table 1.

**Table 1:** Influence of Pd^II^ salts on catalytic performance. 
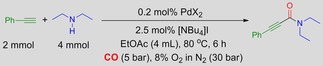

Counterion X^−^	Conv. alkyne^[a]^ [%]	Product yield^[a]^ [%]
I^−^	76	75
Cl^−^	71	66
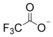	77	73
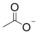	87^[b]^	86^[b]^
	77^[c]^	68^[c]^
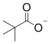	86	84
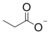	79	73

[a] Conversion and yield were determined by GC using biphenyl as internal standard. All results shown are an average of two experiments. [b] Pd(OAc)_2_ of purity ≥99.9 %. [c] Pd(OAc)_2_ of purity 99 %.

The reaction proceeded with all the anions tested; however, the best results were observed with carboxylate anions such as acetate, and pivalate. This contrasts with what we observed in the previous 2‐alkynoate studies, with catalyst activity only obtained if carboxylate anions were used.^11^ Acetate has previously been shown to be a key component in oxidative carbonylation reactions for the synthesis of 2‐alkynoates, either as the Pd salt or as an additive in the form of NaOAc.^13^, ^24^ The observation that the 2‐ynamide reaction proceeds readily without the presence of carboxylate anions would suggest that the role of the acetate in the 2‐alkynoate reactions is for deprotonation of the alcohol; something previously shown in Pd^II^‐catalysed alcohol oxidation reactions.^25^ We also noted the importance of the Pd(OAc)_2_ salt purity, something that we also observed in the synthesis of 2‐alkynoates. The importance of Pd salt purity and its effect on catalytic performance has been discussed recently,^26^ and we believe it is worth highlighting such factors to aid reproducibility.

Having chosen to utilise Pd(OAc)_2_ for the remainder of the studies, we then went on to study the effect of ligands. For the synthesis of 2‐alkyonates the system was greatly affected by the presence of ligands and we found optimal performance with tetramethylethylenediamine (TMEDA).^11^ However, in these aminocarbonylation studies we found that ligands (such as TMEDA and phenanthroline) were not necessary and did not lead to any enhanced performance under the conditions studied. It could be that in this case the amine substrate acts as an adequate ligand. The lack of ligand and the loading are very similar to the conditions by Gabriele et al., who used 0.2 mol % PdI_2_ (Scheme 1 D).^12^ However, it contrasts the recent work by Xia and co‐workers, stating that under their conditions superior performance was obtained if they employed an *N*‐heterocyclic carbene ligand (Scheme 1 E) and the catalyst loading was 1 mol %.^14^ We were pleased to discover that we could avoid the use of ligands and higher catalyst loadings, clearly an advantage from both economical and green perspectives.

In many previous reports of Pd oxidative carbonylations, iodide salts were a key ingredient; therefore, we examined the effect of such additives, as shown in Table 2.

**Table 2:** Effect of additives on the catalytic system. 
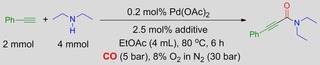

Additive	Conv. alkyne^[a]^ [%]	Product yield^[a]^ [%]
–	24	1
[NBu_4_]I	87	86
[NBu_4_]Br	39	22
[NBu_4_][OAc]	9	3
KI	34	1
KI/[NBu_4_][OAc]^[b]^	9	3

[a] Conversion and yield were determined by GC using biphenyl as internal standard. All results shown are an average of two experiments. [b] 1:1 ratio, 5 mol % overall.

Although KI was used by Gabriele et al. (Scheme 1 D)^12^ and Xia and co‐workers (Scheme 1 E),^14^ it can be seen that we observed little of the desired 2‐ynamide when using KI under our reaction conditions. We presume that the high activity with [NBu_4_]I is the result of increased solubility in our chosen reaction solvent, ethyl acetate. We also found the same behaviour with the previously mentioned 2‐alkynoate system.^11^ We tested various loadings of [NBu_4_]I, comparing 1.25, 2.5 and 5 mol %, to find 2.5 mol % to be the optimum. At present, there is no well‐proven explanation or consensus on the importance of such iodide salts in the oxidative carbonylation reactions, and further work is needed to develop a better understanding.

We tested other parameters of the reaction system, including solvent, base and reaction temperature. The results are shown in Table 3.

**Table 3:** Comparison of reaction system parameters. 
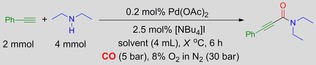

Entry	Solvent	Base^[b]^	Temp. [°C]	Conv. alkyne^[a]^ [%]	Product yield^[a]^ [%]
1	CH_3_CN	–	80	81	53
2	1,4‐dioxane	–	80	75	71
3	EtOAc	–	80	87	86
4	EtOAc	K_3_PO_4_	80	35	32
5	EtOAc	–	60	44	43
6	EtOAc	–	100	90	84
7^[c]^	EtOAc	–	80	93	83
8^[d]^	EtOAc	–	80	52	47

[a] Conversion and yield were determined by GC using biphenyl as internal standard. All results shown are an average of two experiments. [b] 2 equiv. of base were added. [c] Reaction run for 16 h. [d] 1:1 ratio of alkyne/amine.

It was shown that ethyl acetate out‐performed even the commonly employed 1,4‐dioxane under these conditions (Table 3, entries 2 and 3). K_3_PO_4_ was used by Xia and co‐workers (Scheme 1 E);^14^ however, under our conditions we observed that the addition of this base decreased the yield (entry 4). A longer reaction time (entry 7) was also tested; however, the results obtained were almost identical to those after 6 h. We found 80 °C to be the optimal temperature; at 60 °C the activity was greatly reduced, and 100 °C led to a reduction in selectivity. We compared a substrate ratio of 1:1 (entry 8) but found that this caused a significant drop in product yield.

Once the reaction system had been optimised, we tested the system on a variety of alkynes and amines. The results can be seen in Scheme 2. We were able to use both activated and unactivated alkynes. Alkynes containing both electron‐withdrawing and electron‐donating substituents worked well. The system was proven to be heteroatom tolerant, as can be seen with **5** and **6**. Sterically hindered alkynes such as 2‐ethynyl‐1,3,5‐trimethylbenzene **9** afforded a good yield.

**Scheme 2:**
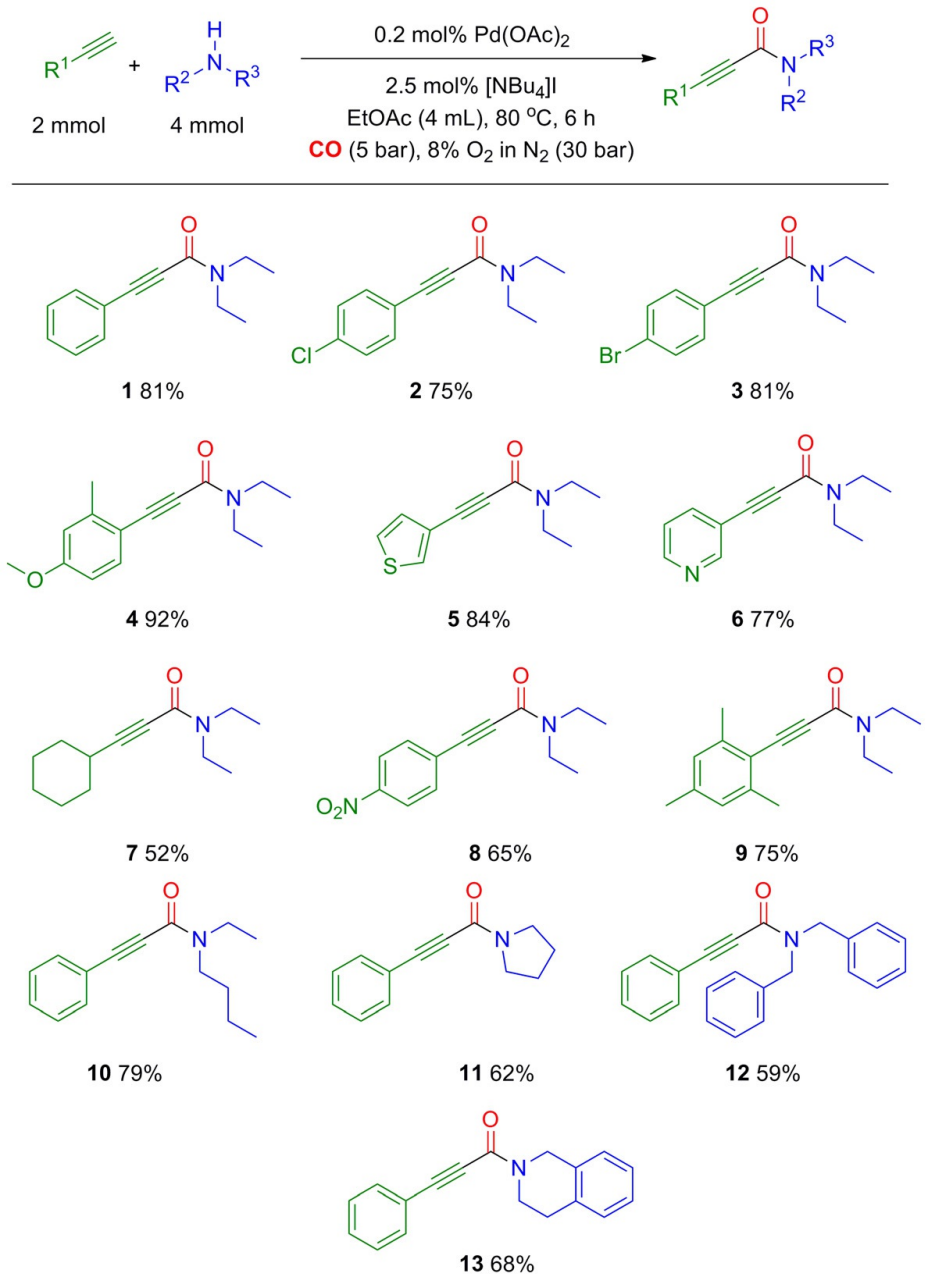
Substrate scope utilising various alkynes and amines. Yields reported are isolated yields. Further details are given in the Supporting Information.

In conclusion, we have developed a method for the synthesis of 2‐ynamides through oxidative carbonylation, which utilises low loadings of Pd^II^ and does not require the use of ligands. Importantly, the method avoids the use of dangerous 1,4‐dioxane, which was previously the solvent of choice for these reactions. The use of [NBu_4_]I enabled us to employ the industrially recommended solvent ethyl acetate. We used O_2_ as the terminal oxidant and demonstrated that the catalyst could operate under safer conditions with low O_2_ concentrations. These factors are important if we are to hope that such catalytic methods are to be exploited on a larger scale.

## Experimental Section

Reactions were performed in 45 mL high‐pressure reactors made of Hastelloy C276 and fitted with a safety pressure‐relief valve. The reaction mixture was placed in a glass liner equipped with a magnetic stirrer. To the glass liner, [NBu_4_]I (2.5 mol %, 0.05 mmol, 0.0185 g) and Pd(OAc)_2_ (0.2 mol %, 0.004 mmol, 0.0009 g) from a stock solution in ethyl acetate (4 mL) were added. This was followed by the addition of alkyne (2 mmol) and amine (4 mmol). The glass liner was placed in a reactor and then pressurized with 5 bar CO, followed by O_2_/N_2_ (8:92) to give a total reaction pressure of 35 bar. The reactor was then stirred on a pre‐heated heating block at 80 °C for 6 h. Once the reaction was complete, the reactor body was cooled in an ice‐bath and then slowly depressurised in a fume hood. The reaction mixture was then poured into a separating funnel, and brine was added. The aqueous layer was separated and back‐extracted with ethyl acetate twice. The combined organic layers were dried over magnesium sulfate, filtered and concentrated under reduced pressure. The product was purified by silica gel flash column chromatography, and the appropriate fractions were combined and concentrated under reduced pressure. The product was then dried under high vacuum.

Note: Appropriate safety precautions should be in place when performing these reactions. More experimental details and analytical data are given in the Supporting Information.

## Supporting information

As a service to our authors and readers, this journal provides supporting information supplied by the authors. Such materials are peer reviewed and may be re‐organized for online delivery, but are not copy‐edited or typeset. Technical support issues arising from supporting information (other than missing files) should be addressed to the authors.

Supplementary
