# Supplementary material for: Palladium(II)‐Catalysed Aminocarbonylation of Terminal Alkynes for the Synthesis of 2‐Ynamides: Addressing the Challenges of Solvents and Gas Mixtures
Source: ChemSusChem. 2017 Jan 23;10(4):675–80. doi: 10.1002/cssc.201601601 (PMC5347853; doi:10.1002/cssc.201601601)
Supplement: Supplementary file 1 — Supplementary [file CSSC-10-675-s001.pdf]

CHEMISTRY & SUSTAINABILITY
CHEMSUSCHEM
ENERGY & MATERIALS

Supporting Information

Palladium(II)-Catalysed Aminocarbonylation of Terminal Alkynes for the Synthesis of 2-Ynamides: Addressing the Challenges of Solvents and Gas Mixtures

N. Louise Hughes, Clare L. Brown, Andrew A. Irwin, Qun Cao, and Mark J. Muldoon*[a]

cssc_201601601_sm_miscellaneous_information.pdf

Supporting Information

Palladium(II) Catalysed Aminocarbonylation of Terminal Alkynes for the Synthesis of 2-Ynamides: Addressing the Challenges of Solvents and Gas Mixtures

N. Louise Hughes, Clare L. Brown, Andrew A. Irwin, Qun Cao and Mark J. Muldoon*

Queen's University Belfast, m.j.muldoon@qub.ac.uk

1. General Information

Unless otherwise stated, all reagents were purchased from Sigma-Aldrich and used without further purification. The following chemicals were purchased from Fluorochem: 4-chlorophenylacetylene, 4-bromophenylacetylene, 4-cyanophenylacetylene, ethynylcyclohexane, 1-ethynyl-4-nitrobenzene, and palladium(II) acetate (99%). The following chemicals were purchased from Alfa Aesar: palladium(II) trifluoroacetate (97%), palladium(II) trimethylacetate (97%) and dibenzylamine. The following palladium salts were purchased from Sigma Aldrich: palladium(II) propionate (≥ 99.5%), palladium(II) acetate (≥99.9 trace metal basis), palladium(II) chloride (≥99.9%), palladium(II) iodide (≥99.9%).

Thin layer chromatography (TLC) was carried out using Merck TLC silica gel 60 sheets, and visualized with ultraviolet light or potassium permanganate stain. Flash column chromatography (FCC) was performed with Fluorochem silica gel 60 Å as the stationary phase and solvents employed were analytical grade. ^{1}H NMR spectra were recorded on a Bruker AVX400 (400 MHz) spectrometer at ambient temperature. ^{13}C NMR spectra were recorded on a Bruker AVX400 (100 MHz) spectrometer at ambient temperature. Mass spectra (ESI) data were analyzed using Waters LCT Premier TOF. Melting points were measured on Stuart melting point apparatus (Digital, SMP10). IR spectra were measured on PerkinElmer Spectrum 100 FT-IR Spectrometers. Carbon monoxide (CP Grade), air and O_2 cylinders were from BOC and pre-mixed O_2:N_2(8:92) (β standard) cylinder was from BOC Special Gases.

Gas chromatography analysis was carried out using Agilent 7820A series gas chromatograph. An Agilent 19091J-413HP-5 column (30.0 m x 320 µm x 0.25 µm nominal) was employed for all the separations using the following conditions: initial column temperature, 40 °C; initial hold time, 1 min; next temperature, 100 °C; hold time, 5 min; rate of temperature ramp 2, 30 °C/ min; injection temperature, 250 °C; injection volume 1 µL; detection temperature, 300 °C split mode. The effluent was combusted in an H_2/ air flame and detected using FID (flame ionization detector).

The GC yield of products and conversion of alkyne were determined by using an internal standard (biphenyl). The response factor (R_f) of an analyte was determined by analysing known quantities of internal standard (biphenyl) against known quantities of substrate and product according to the following equation:

$$RF = \frac{Area_{internal\ standard} \times Moles_{analyte}}{Area_{analyte} \times Moles_{internal\ standard}}$$

The quantity of an analyte was then determined by the following equation:

$$Moles_{analyte} = \frac{RF \times Moles_{internal\ standard} \times Area_{analyte}}{Area_{internal\ standard}}$$

2. Experimental

Safety Considerations

Catalytic oxidative carbonylations should be carried out by trained personnel, with suitable safety measures and utilizing appropriate equipment. Suitable precautions should be taken by those wishing to reproduce or extend this type of work.

In these studies, high pressure O_2 gas mixtures (air and 8% O_2) are employed with organic solvents and CO. We use pressures which are significantly below the pressure ratings of the vessels, and vessels are equipped with safety relief valves (set to release pressure at 100 bar). Carbon monoxide is a flammable and highly toxic gas. The CO cylinder was stored in a ventilated cylinder cupboard adjacent to the fume hood. A CO monitor / alarm was used in order to detect any leaks. Pressurized tubing and reactors were all vented in the fume hood.

General notes

$Pd(OAc)_2$ which was ≥99.9% trace metal basis purity (from Sigma Aldrich) was used and it was found that lower grades of $Pd(OAc)_2$ led to reduced yields. (See Table 1 in paper for more information) $Pd(OAc)_2$ was added to the reactions *via* stock solution. Stock solutions were kept for a maximum of three days.

Reactions were all carried out in pressure vessels which were heated and stirred on a hotplate stirrer. Reactions were carried out in glass liners and stirred using Teflon coated magnetic stirrer bars. If reactions were carried out without using a glass liner, it was found that the Hastelloy C276 reactor body caused a dramatic reduction in the yield of product produced (and selectivity).

2.1 General Method for Optimisation of Catalytic System

Reactions were performed in 45 mL high-pressure reactors made of hastelloy and the reaction mixture was placed in a glass liner, equipped with a magnetic stirrer.

To the glass liner, tetrabutylammonium iodide (2.5 mol%, 0.05 mmol, 0.0185 g) and $Pd(OAc)_2$ (0.2 mol%, 0.004 mmol, 0.0009 g) from a stock solution in ethyl acetate (4 mL) were added. This was followed by the addition of alkyne (2 mmol) and amine (4 mmol). The glass liner was placed in a reactor and then pressurized

with 5 bar of carbon monoxide gas, followed by O_2:N_2 (8:92) to give a total reaction pressure of 35 bar. The reactor was then stirred on a pre-heated heating block at 80 °C for six hours. Once the reaction was complete, the reactor was cooled in an ice bath and slowly depressurised in a fume hood. Internal standard (biphenyl) (~0.2 g) was added, and the glass liner magnetically stirred for 1 minute to ensure all standard was fully dissolved. A sample was then prepared for GC analysis by filtration through a silica plug with diethyl ether to remove any catalyst components. The sample was then submitted for GC analysis.

2.2 General Method for Preparation of Isolated Substrates

Procedure was the same as described above, however at the end of the reaction the reactor was cooled and depressurized, then poured into a separating funnel and brine added. The aqueous layer was then separated and back extracted with ethyl acetate twice. The combined organic layers were dried over magnesium sulphate, filtered and concentrated under reduced pressure. The product was purified by silica gel flash column chromatography, and the appropriate fractions combined and concentrated under reduced pressure. The product was then dried under high vacuum.

3. Product Characterization Data

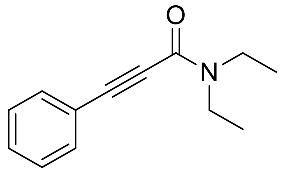

***N,N*-diethyl-3-phenylpropiolamide (1)**

Purified by flash column chromatography (0.1 % Et_3N in hexane / ethyl acetate 2:1) to afford **1** as a golden orange oil (0.3325 g, 81 %). ^1H NMR (400 MHz, $CDCl_3$): δ 7.47-7.44 (m, 2H), 7.35-7.25 (m, 3H), 3.62-3.55 (q, *J*= 7.1 Hz, 2H), 3.43-3.36 (q, *J*= 7.1 Hz, 2H), 1.23-1.17 (t, *J*= 7.1 Hz, 3H), 1.12-1.07 (t, *J*= 7.2 Hz, 3H). ^{13}C NMR (101 MHz, $CDCl_3$): δ 154.3, 132.7, 130.2, 128.9, 121.1, 89.3, 82.3, 44.0, 39.7, 14.8, 13.2. HRMS (ESI^+) Calc. for $C_{26}H_{31}N_2O_2$ [$2M+H^+$] 403.2386, found: 403.2384. IR (neat): 2988, 2225, 1617, 1424, 1286, 1136, 757, 689 cm^{-1}. Analytical data was in correspondence with literature data.[1]

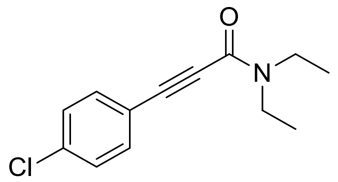

3-(4-chlorophenyl)-*N,N*-diethylpropiolamide (2)

Purified by flash column chromatography (0.1 % Et_3N in hexane / ethyl acetate 7:3) to afford **2** as an orange crystalline solid (0.3560 g, 75 %). ^1H NMR (400 MHz, $CDCl_3$): δ 7.48-7.44 (m, 2H), 7.36-7.32 (m, 2H), 3.68-3.61 (q, *J*= 7.1 Hz, 2H), 3.51-3.44 (q, *J*= 7.2 Hz, 2H), 1.30-1.25 (t, *J*= 7.2 Hz, 3H), 1.21-1.16 (t, *J*= 7.2 Hz, 3H). ^{13}C NMR (101 MHz, $CDCl_3$): δ 153.9, 136.3, 133.7, 129.1, 119.4, 87.9, 82.9, 43.7, 39.5, 14.6, 13.0. HRMS (ESI^+) Calc. for $C_{26}H_{29}N_2O_2Cl_2$ [$2M+H^+$] 471.1606, found: 471.1625. m.p. 71 °C. IR (neat): 2991, 2217, 1610, 1430, 1298, 1088, 840, 733 cm^{-1}. Analytical data was in correspondence with literature data.[1]

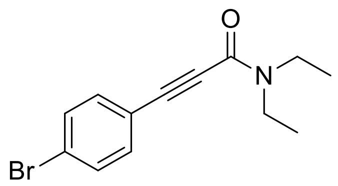

3-(4-bromophenyl)-*N,N*-diethylpropiolamide (3)

Purified by flash column chromatography (0.1 % Et_3N in hexane / ethyl acetate 2:1) to afford **3** as a light brown solid (0.4499 g, 81 %). ^1H NMR (400 MHz, $CDCl_3$): δ 7.53 - 7.49 (m, 2H), 7.41-7.37 (m, 2H), 3.68-3.61 (q, *J*= 7.2 Hz, 2H), 3.51-3.44 (q, *J*= 7.2 Hz,

2H), 1.31-1.24 (t, *J*= 7.2 Hz, 3H), 1.21-1.15 (t, *J*= 7.2 Hz, 3H). ^{13}C NMR (101 MHz, $CDCl_3$): δ 153.9, 133.8, 132.0, 124.1, 119.8, 87.9, 83.0, 43.7, 39.5, 14.6, 13.0. HRMS (ESI^+) Calc. for $C_{13}H_{15}NOBr$ [$M+H^+$] 280.0337, found: 280.0333. m.p. 93-94 °C. IR (neat): 2995, 2217, 1611, 1431, 1292, 1008, 836, 733 cm^{-1}. Analytical data was in correspondence with literature data.[1]

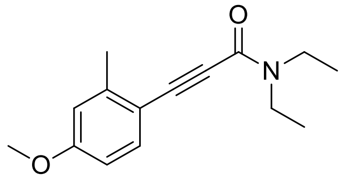

***N, N*-diethyl-3-(4-methoxy-2-methylphenyl)propiolamide (4)**

Purified by flash column chromatography (0.1 % Et_3N in hexane / ethyl acetate 3:2) to afford **4** as an orange crystalline solid (0.4525 g, 92 %). ^{1}H NMR (400 MHz, $CDCl_3$): δ 7.48-7.44 (d, *J*= 8.5 Hz, 1H), 6.77-6.74 (m, 1H), 6.73-6.69 (m, 1H), 3.83-3.80 (s, 3H), 3.70-3.64 (q, *J*= 7.2 Hz, 2H), 3.51-3.45 (q, *J*= 7.2 Hz, 2H), 2.46-2.45 (s, 3H), 1.30-1.26 (t, *J*= 7.2 Hz, 3H), 1.21-1.16 (t, *J*= 7.2 Hz, 3H). ^{13}C NMR (101 MHz, $CDCl_3$): δ 160.9, 154.5, 143.4, 134.8, 115.4, 112.9, 111.7, 88.7, 85.1, 55.4, 43.7, 39.4, 21.1, 14.6, 13.1. HRMS (ESI^+) Calc. for $C_{30}H_{39}N_2O_4$ [$2M+H^+$] 491.2910, found: 491.2912. m.p. 40-41 °C. IR (neat): 2980, 2214, 1606, 1425, 1289, 1248, 1113, 865, 730 cm^{-1}.

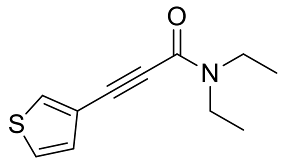

***N,N*-diethyl-3-(thiophen-3-yl)propiolamide (5)**

Purified by flash column chromatography (0.1 % Et_3N in hexane / ethyl acetate 3:2) to afford **5** as a golden oil (0.3667 g, 84 %). ^{1}H NMR (400 MHz, $CDCl_3$): δ 7.66-7.62 (d, *J*= 1.7 Hz, 1H), 7.33-7.29 (dd, *J*= 3.2 Hz, 4.7 Hz, 1H), 7.21-7.17 (d, *J*= 4.1 Hz, 1H), 3.70-3.60 (q, *J*= 7.1 Hz, 2H), 3.51-3.42 (q, *J*= 7.1 Hz, 2H), 1.31-1.23 (t, *J*= 7.1 Hz, 3H), 1.21-1.13 (t, *J*= 7.2 Hz, 3H). ^{13}C NMR (101 MHz, $CDCl_3$): δ154.1, 131.9, 130.1, 126.0, 120.1, 84.6, 82.0, 43.7, 39.4, 14.5, 13.0. HRMS (ESI^+) Calc. for $C_{22}H_{27}N_2O_2S_2$ [$2M+H^+$] 415.1514, found: 415.1524. IR (neat): 3092, 2980, 2221, 1611, 1428, 1277, 1130, 784, 626 cm^{-1}.

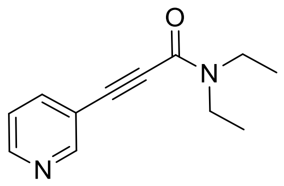

***N,N*-diethyl-3-(pyridin-3-yl)propiolamide (6)**

Purified by flash column chromatography (0.1 % Et_3N in ethyl acetate / hexane 3:1) to afford **6** as a yellow oil (0.3051 g, 77 %). ^1H NMR (400 MHz, $CDCl_3$): δ 8.78-8.73 (m, 1H), 8.65-8.60 (dd, *J*= 4.9 Hz, 1.7 Hz, 1H), 7.86-7.81 (m, 1H), 7.34-7.29 (m, 1H), 3.70-3.62 (q, *J*= 7.2 Hz, 2H), 3.53-3.45 (q, *J*= 7.2 Hz, 2H), 1.32-1.27 (t, *J*= 7.2 Hz, 3H), 1.22-1.15 (t, *J*= 7.2 Hz, 3H). ^{13}C NMR (101 MHz, $CDCl_3$): δ 153.5, 152.8, 150.2, 139.4, 123.3, 118.2, 85.6, 85.0, 43.8, 39.5, 14.6, 13.0. HRMS (ESI^+) Calc. for $C_{36}H_{42}N_6O_3Na$ [3M+Na$^+$] 629.3216, found: 629.3199. IR (neat): 2987, 2221, 1615, 1428, 1289, 1140, 703, 580 cm^{-1}. Analytical data was in correspondence with literature data.[1]

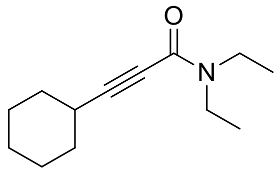

3-cyclohexyl-*N,N*-diethylpropiolamide (7)

Purified by flash column chromatography (0.1 % Et_3N in hexane / ethyl acetate 3:1) to afford **7** as a golden oil (0.2201 g, 52 %). ^1H NMR (400 MHz, $CDCl_3$): δ 3.61-3.54 (q, *J*= 7.1 Hz, 2H), 3.45-3.37 (q, *J*= 7.2 Hz, 2H), 2.60-2.51 (m, 1H), 1.87-1.78 (m, 2H), 1.76-1.66 (m, 2H), 1.58-1.46 (m, 3H), 1.41-1.28 (m, 3H), 1.24-1.17 (t, *J*= 7.1 Hz, 3H), 1.15-1.10 (t, *J*= 7.2 Hz, 3H). ^{13}C NMR (101 MHz, $CDCl_3$): δ154.4, 95.3,74.4,43.6, 39.2, 31.8, 29.1, 25.8, 24.7, 14.3, 13.0. HRMS (ESI^+) Calc. for $C_{26}H_{43}N_2O_2$ [2M+H$^+$] 415.3324, found: 415.3314. IR (neat): 2930, 2232, 1621, 1423, 1275, 1222, 1170, 1087, 894, 737 cm^{-1}.

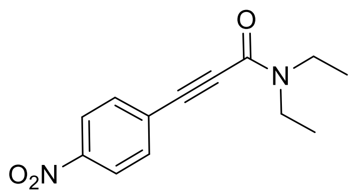

***N,N*-diethyl-3-(4-nitrophenyl)propiolamide (8)**

Purified by flash column chromatography (0.1 % Et_3N in hexane / ethyl acetate 3:2) to afford **8** as a dark red crystalline solid (0.3158 g, 65 %). ^1H NMR (400 MHz, $CDCl_3$): 8.26-8.21 (m, 2H), 7.72-7.66 (m, 2H), 3.70-3.63 (q, *J*= 7.2 Hz, 2H), 3.53-3.46 (q, *J*= 7.2 Hz, 2H), 1.33-1.26 (t, *J*= 7.2 Hz, 3H), 1.22-1.18 (m, 3H). ^{13}C NMR (101 MHz, $CDCl_3$): δ 153.2, 148.7, 133.2, 127.6, 125.7, 124.1, 123.9, 86.4, 86.1, 43.8, 39.6, 14.6, 12.9. m.p. 90 °C. HRMS (ESI^+) Calc. for $C_{26}H_{29}N_4O_6$ [2M+H$^+$] 493.2087, found:

493.2069. IR (neat): 3034, 2221, 1623, 1420, 1191, 755, 690 cm^{-1}. Analytical data was in correspondence with literature data.[2]

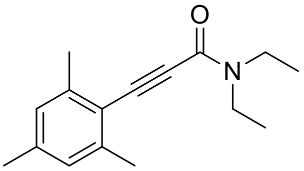

_N,N_-diethyl-3-mesitylpropiolamide (9)

Purified by flash column chromatography (0.1 % Et_3N in hexane / ethyl acetate 3:1) to afford **9** as a golden oil (0.3654 g, 75 %). ^1H NMR (400 MHz, $CDCl_3$): δ 6.91-6.85 (s, 2H), 3.74-3.62 (q, *J*= 7.1 Hz, 2H), 3.52-3.43 (q, *J*= 7.1 Hz, 2H), 2.47-2.40 (s, 6H), 2.32-2.24 (s, 3H), 1.30-1.24 (t, *J*= 7.1 Hz, 3H), 1.22-1.16 (t, *J*= 7.1 Hz, 3H). ^{13}C NMR (101 MHz, $CDCl_3$): δ 154.4, 141.6, 139.8, 128.2, 127.9, 117.7, 89.7, 87.4, 43.6, 39.4, 21.5, 21.0, 14.6, 13.0. HRMS (ESI^+) Calc. for $C_{32}H_{43}N_2O_2$ [$2M+H^+$] 487.3325, found: 487.3336. IR (neat): 2984, 2206, 1621, 1417, 1280, 1128, 852, 733 cm^{-1}.

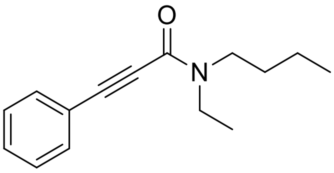

_N_-butyl-_N_-ethyl-3-phenylpropiolamide (10)

Purified by flash column chromatography (0.1 % Et_3N in hexane / ethyl acetate 3:1) to afford **10** as a golden yellow oil (0.3609 g, 79%). ^1H NMR (400 MHz, $CDCl_3$): δ 7.56-7.50 (m, 2H), 7.44-7.32(m, 3H), 3.70-3.58 (m, 2H), 3.50-3.39 (m, 2H), 1.69-1.53 (m, 2H), 1.46-1.31 (m, 2H), 1.30-1.15 (m, 3H), 1.10-0.92 (m, 3H). ^{13}C NMR (101 MHz, $CDCl_3$): δ 154.3, 132.4, 130.0, 128.6, 121.0, 89.2, 89.1, 82.3, 82.1, 48.8, 44.0, 39.8, 31.2, 29.9, 20.3, 20.1, 14.4, 14.0, 12.8. Additional peaks in the carbon NMR are attributed to the presence of rotamers. HRMS (ESI^+) Calc. for $C_{30}H_{38}N_2O_2Na$ [$2M+Na^+$] 481.2831, found: 481.2820. IR (neat): 2966, 2225, 1620, 1423, 1293, 1137, 757, 689 cm^{-1}.

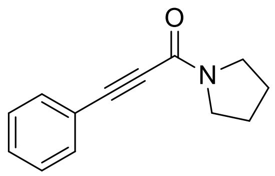

3-phenyl-1-(pyrrolidin-1-yl)prop-2-yn-1-one (11)

Purified by flash column chromatography (hexane / ethyl acetate 1:1) to afford **11** as a dark orange solid (0.2481 g, 62 %). ^{1}H NMR (400 MHz, $CDCl_3$): δ 7.56-7.52 (m, 2H), 7.44-7.33 (m, 3H), 3.77-3.70 (m, 2H), 3.56-3.51 (m, 2H), 2.00-1.93 (m, 4H). ^{13}C NMR (101 MHz, $CDCl_3$): δ 152.9, 132.6, 130.1, 128.6, 120.8, 88.8, 82.8, 48.3, 45.5, 25.6, 24.9. m.p. 65 °C. HRMS (ESI$^+$) Calc. for $C_{26}H_{26}N_2O_2Na$ [2M+Na$^+$] 421.1892, found: 421.1879. IR (neat): 2980, 2221, 1621, 1487, 1416, 1189, 760, 690 cm^{-1}. Analytical data was in correspondence with literature data.[3]

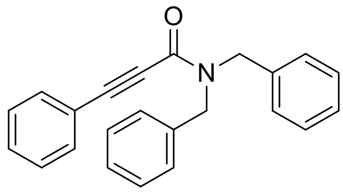

***N,N*-dibenzyl-3-phenylpropiolamide (12)**

Purified by flash column chromatography (0.1 % Et_3N in hexane / ethyl acetate 10:1) to afford **12** as an orange solid (0.4001 g, 59 %). ^{1}H NMR (400 MHz, $CDCl_3$): δ 7.52 - 7.48 (m, 2H), 7.43 -7.26 (m, 13H), 4.78-4.74 (s, 2H), 4.58-4.55 (s, 2H). ^{13}C NMR (101 MHz, $CDCl_3$): δ 155.2, 136.4, 136.2, 132.6, 130.3, 129.0, 128.9, 128.7, 128.6, 128.1, 127.9, 127.8,120.5, 91.0, 81.8, 51.6, 46.5. m.p. 104 °C. HRMS (ESI$^+$) Calc. for $C_{23}H_{20}NO$ [M+H$^+$] 326.1545, found: 326.1536. IR (neat): 3024, 2214, 1621, 1449, 1192, 754, 690, 561 cm^{-1}.

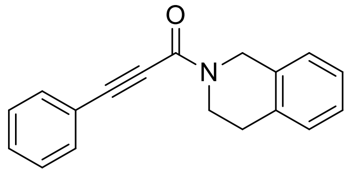

1-(3,4-dihydroisoquinolin-2(1H)-yl)-3-phenylprop-2-yn-1-one (13)

Purified by flash column chromatography (0.1 % Et_3N in hexane / ethyl acetate 7:3) to afford **13** as a light brown solid (0.3535 g, 68 %). ^{1}H NMR (400 MHz, $CDCl_3$): δ 7.61-7.55 (d, *J*=7.8 Hz, 2H), 7.46-7.35 (m, 3H), 7.25-7.13 (m,4H), 5.01-4.78 (m, 2H), 4.10-3.88 (m, 2H), 3.01-2.88 (m, 2H). ^{13}C NMR (101 MHz, $CDCl_3$): δ 153.8, 153.5, 134.7, 134.0, 132.5, 132.4 130.2, 129.1, 128.8, 128.7, 127.2, 126.9, 126.8, 126.7, 126.3, 120.6, 91.2, 90.5, 81.6, 81.5, 48.8, 44.8, 44.2, 39.8, 29.7, 28.5. Additional peaks in the carbon NMR are attributed to the presence of rotamers. m.p. 72-73 °C. HRMS (ESI$^+$) Calc. for $C_{18}H_{15}NONa$ [M+Na$^+$] 284.1051, found: 284.1038. IR (neat): 3066, 2904, 2850, 2225, 1619, 1435, 1193, 741, 691 cm^{-1}. Analytical data was in correspondence with literature data.[4]

4. NMR Spectra

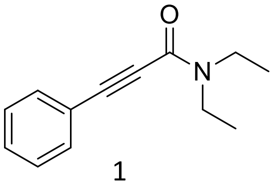

1

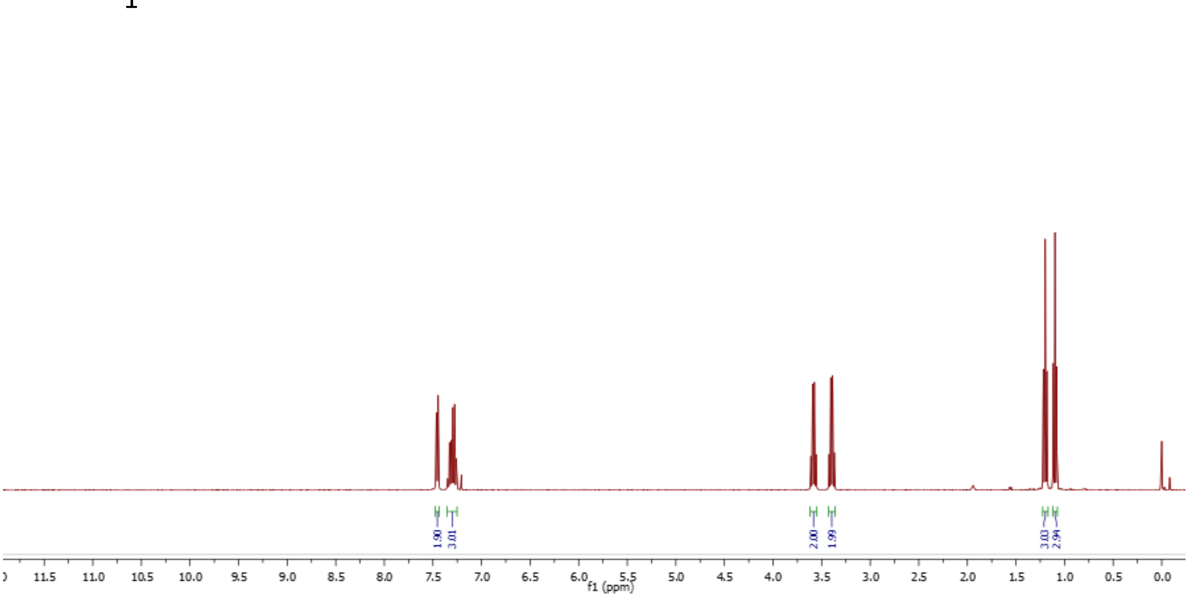

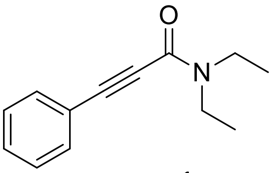

1

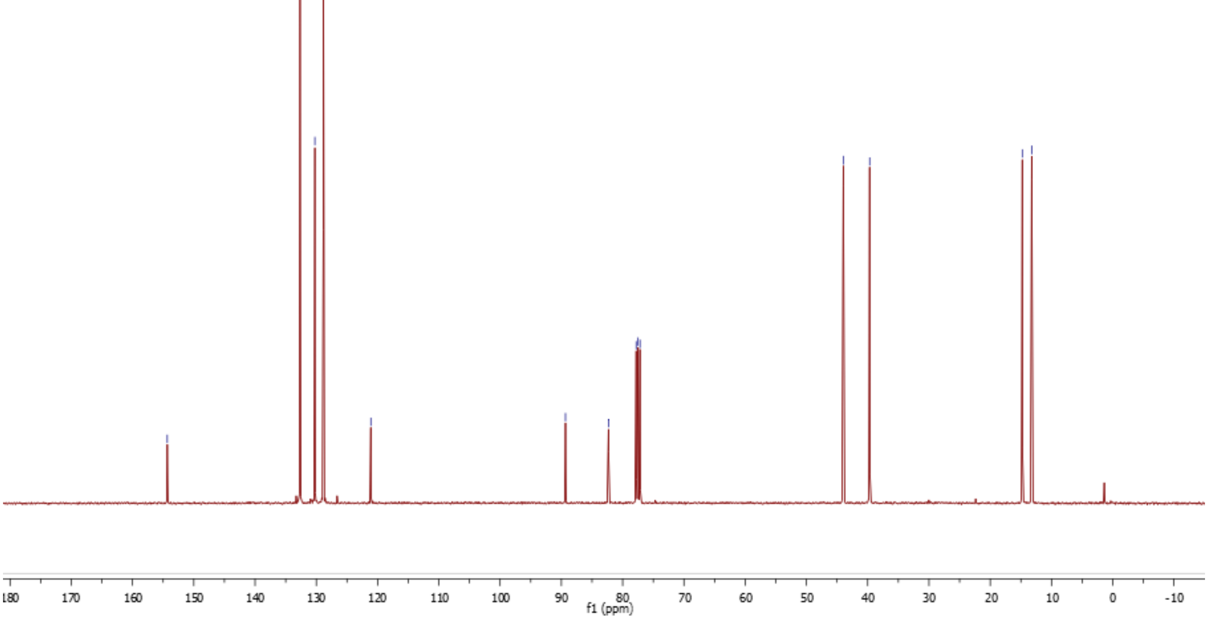

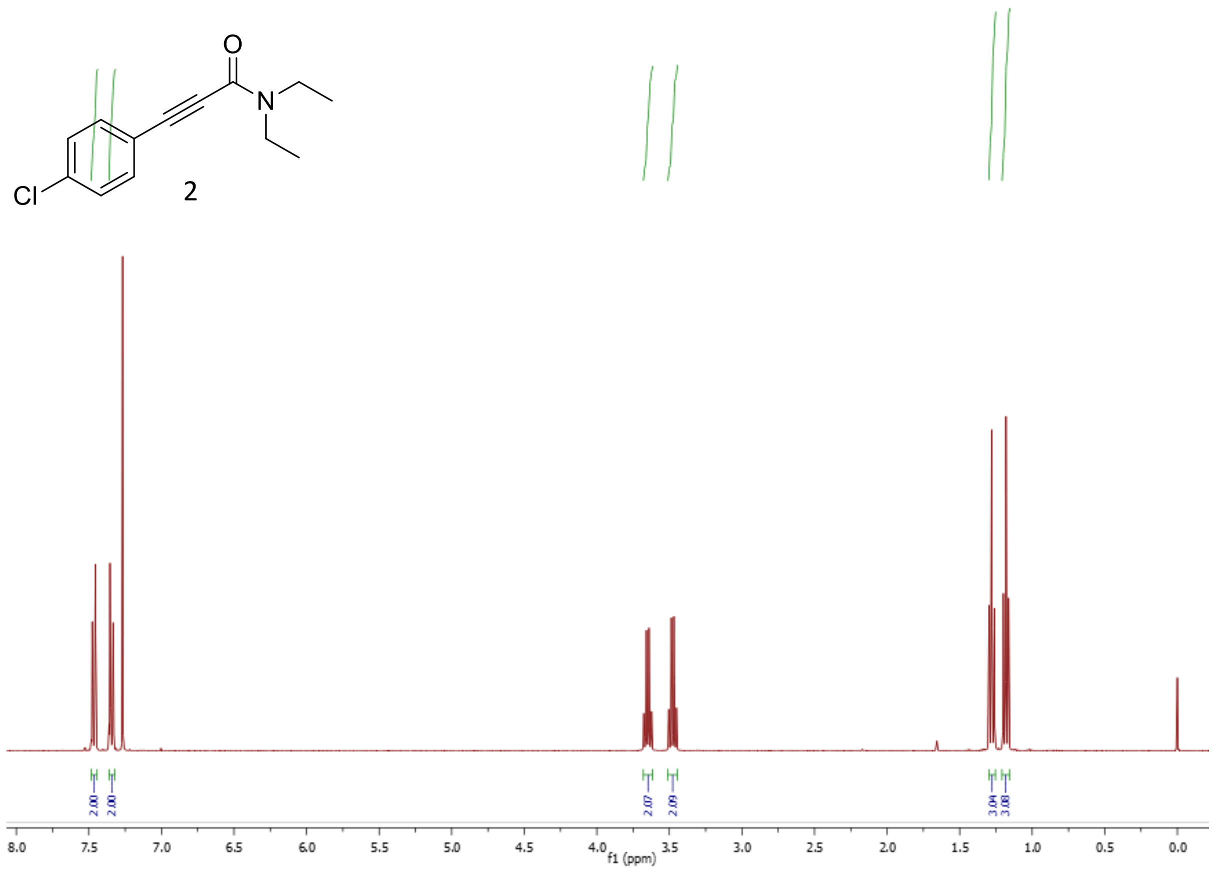
O
N
Cl
2
2.00
2.00
2.07
2.09
3.04
3.08
8.0
7.5
7.0
6.5
6.0
5.5
5.0
4.5
4.0
3.5
3.0
2.5
2.0
1.5
1.0
0.5
0.0
f1 (ppm)

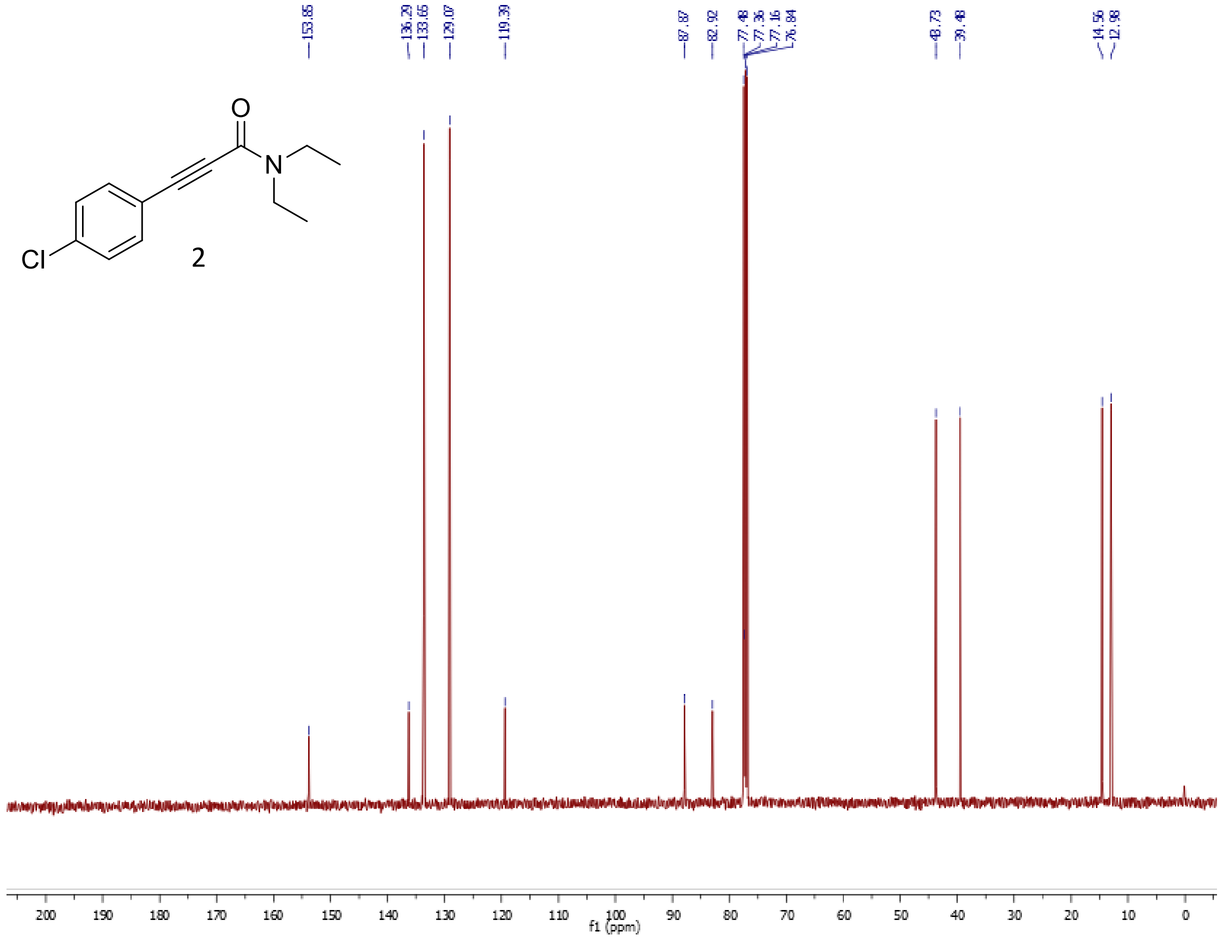
153.85
136.29
133.65
129.07
119.39
87.87
82.92
77.48
77.36
77.16
76.84
43.73
39.48
14.56
12.98
O
N
Cl
2
200
190
180
170
160
150
140
130
120
110
100
90
80
70
60
50
40
30
20
10
0
f1 (ppm)

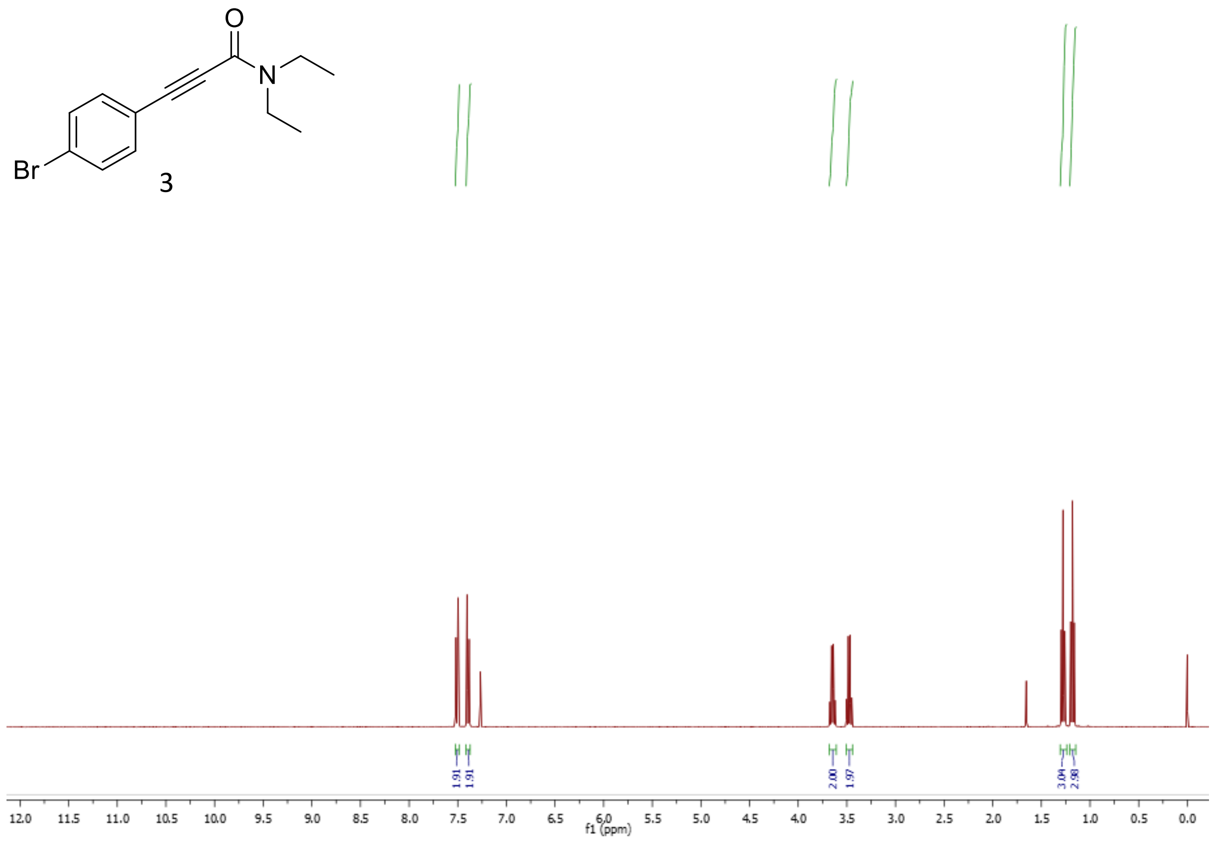

O
N
Br
3
1.91
1.91
2.00
1.97
3.04
2.98
12.0 11.5 11.0 10.5 10.0 9.5 9.0 8.5 8.0 7.5 7.0 6.5 6.0 5.5 5.0 4.5 4.0 3.5 3.0 2.5 2.0 1.5 1.0 0.5 0.0
f1 (ppm)

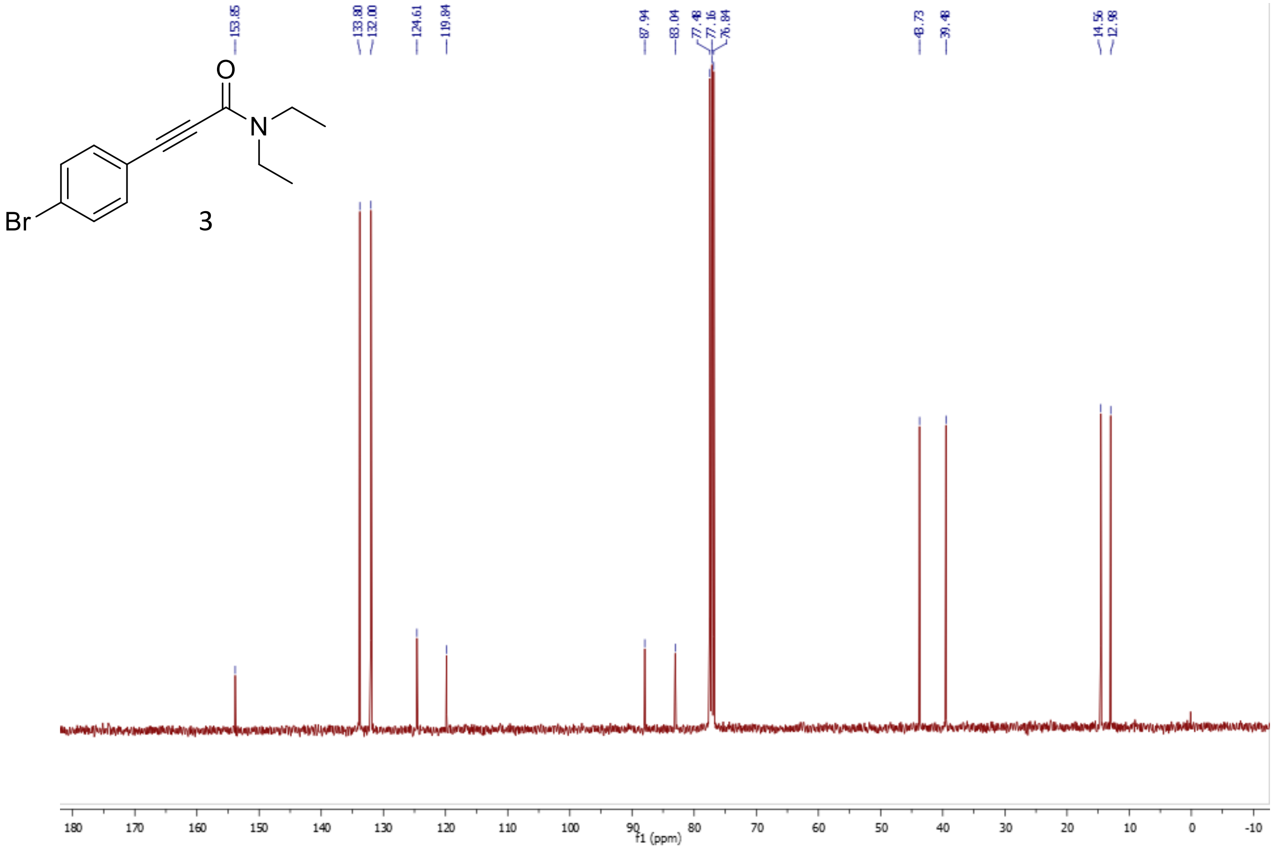

153.85
133.80
132.00
124.61
119.84
87.94
83.04
77.48
77.16
76.84
43.73
39.48
14.56
12.98
O
N
Br
3
180 170 160 150 140 130 120 110 100 90 80 70 60 50 40 30 20 10 0 -10
f1 (ppm)

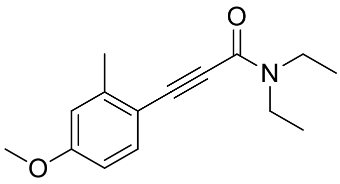

4

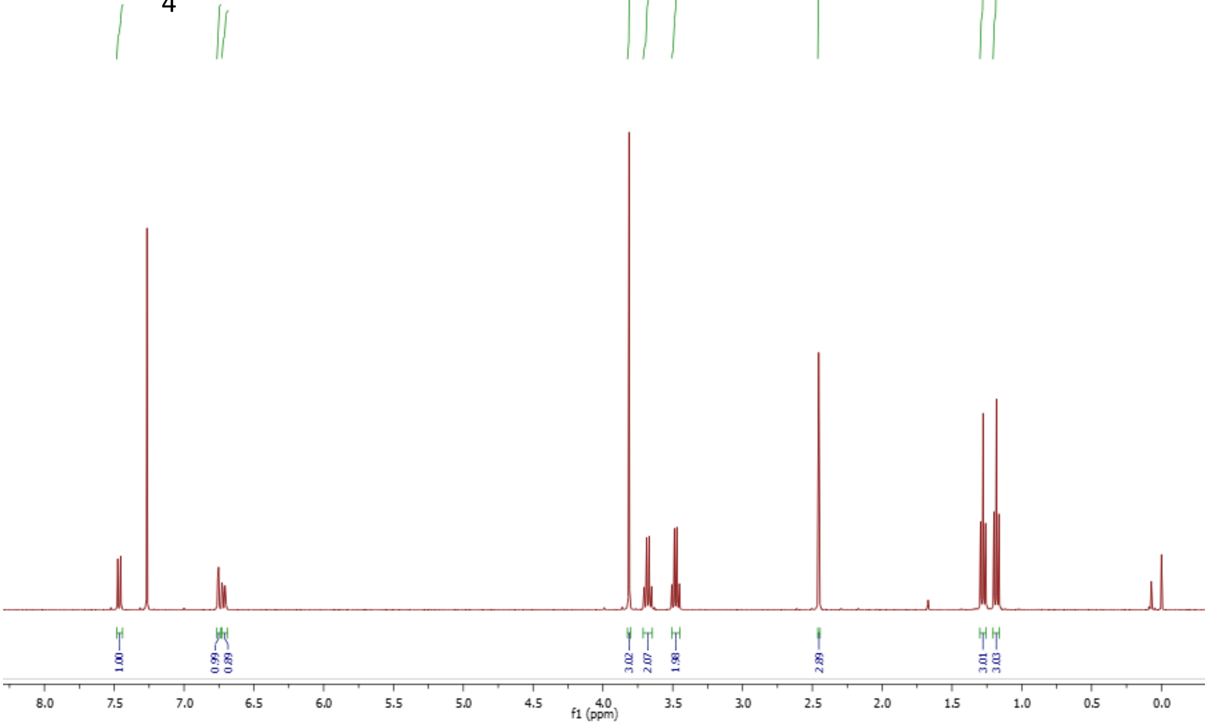
1.00
0.99
0.89
3.02
2.07
1.98
2.89
3.01
3.03
8.0 7.5 7.0 6.5 6.0 5.5 5.0 4.5 4.0 3.5 3.0 2.5 2.0 1.5 1.0 0.5 0.0
f1 (ppm)

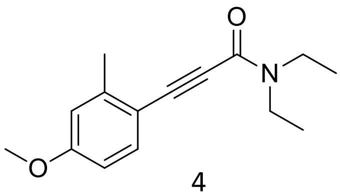

4

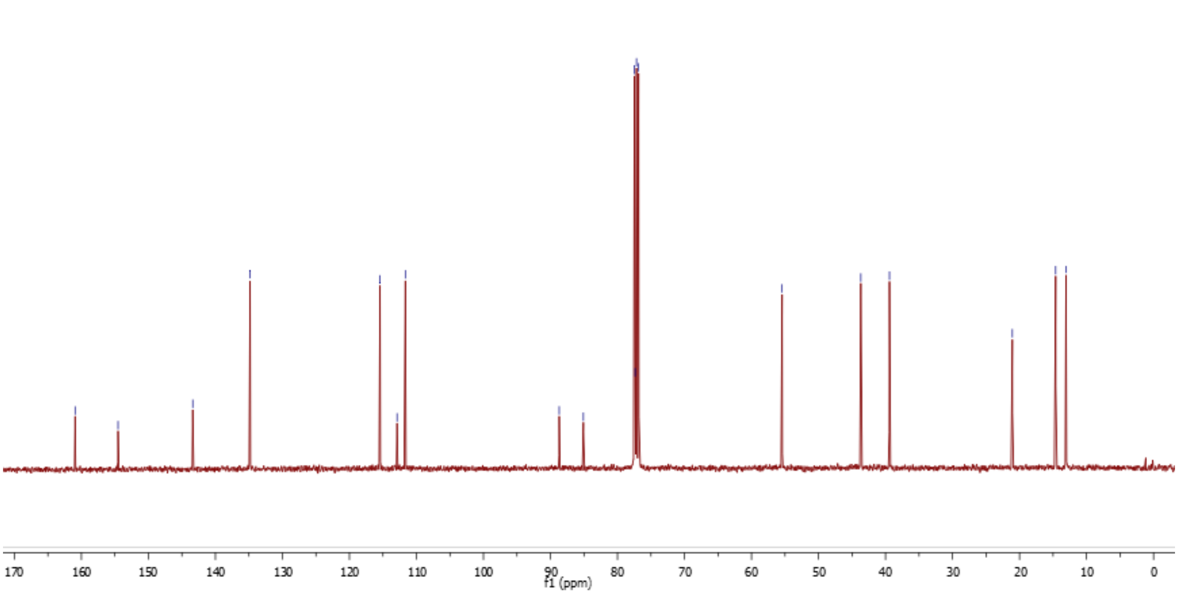
160.94
154.53
143.38
134.82
115.43
112.89
111.66
88.70
85.09
77.48
77.36
77.16
76.84
55.43
43.68
39.41
21.07
14.61
13.06
170 160 150 140 130 120 110 100 90 80 70 60 50 40 30 20 10 0
f1 (ppm)

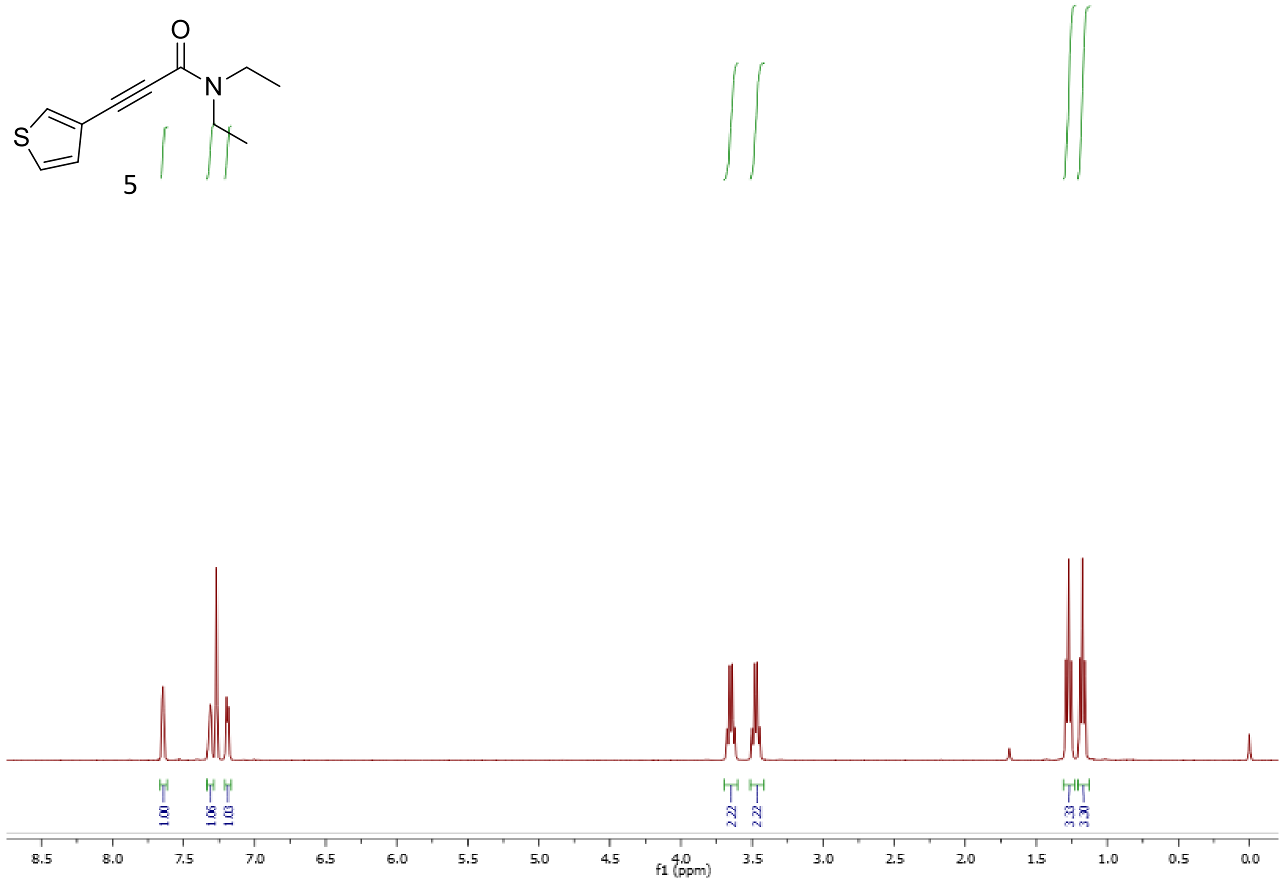
O
N
S
5
1.00
1.06
1.03
2.22
2.22
3.33
3.30
8.5 8.0 7.5 7.0 6.5 6.0 5.5 5.0 4.5 4.0 3.5 3.0 2.5 2.0 1.5 1.0 0.5 0.0
f1 (ppm)

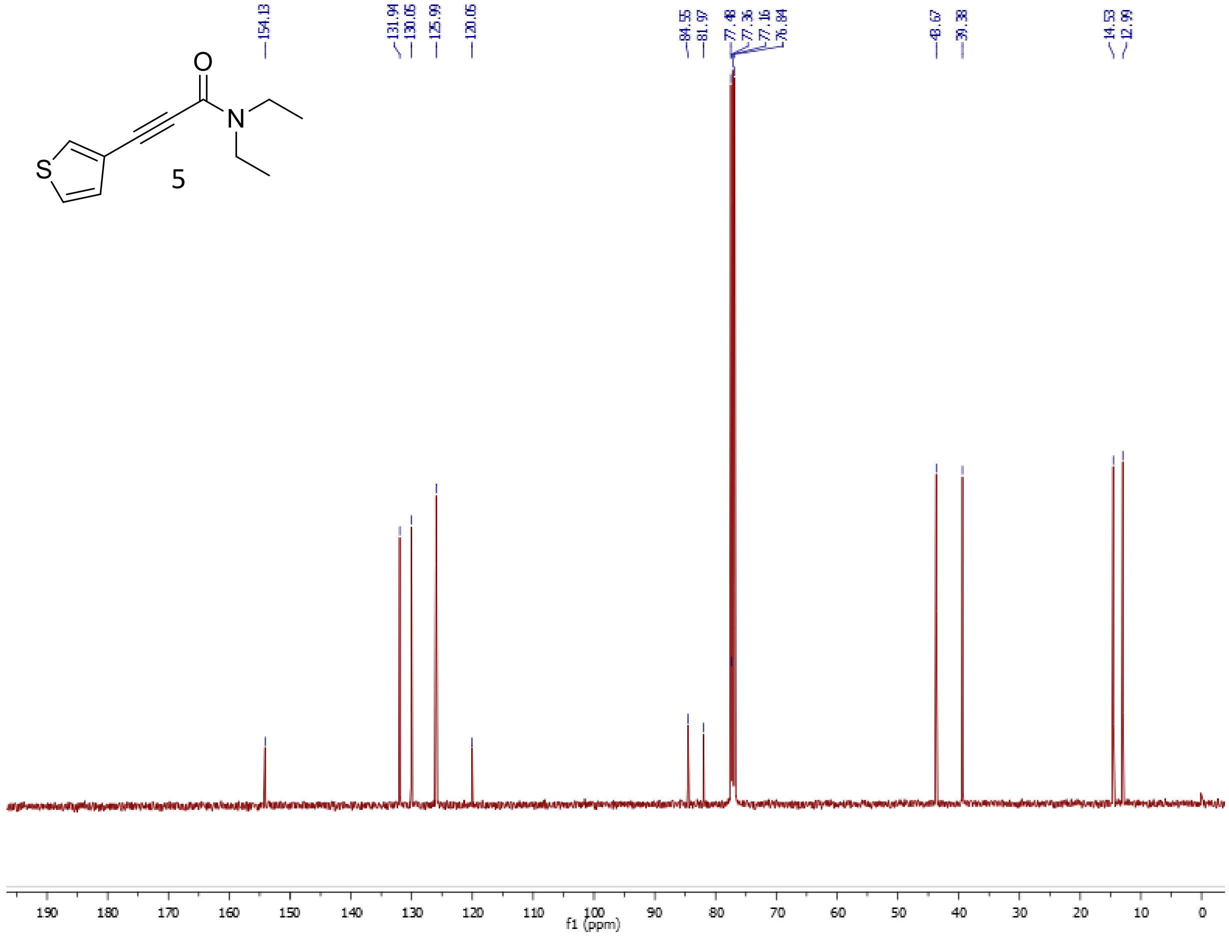
154.13
131.94
130.05
125.99
120.05
84.55
81.97
77.48
77.36
77.16
76.84
43.67
39.38
14.53
12.99
O
N
S
5
190 180 170 160 150 140 130 120 110 100 90 80 70 60 50 40 30 20 10 0
f1 (ppm)

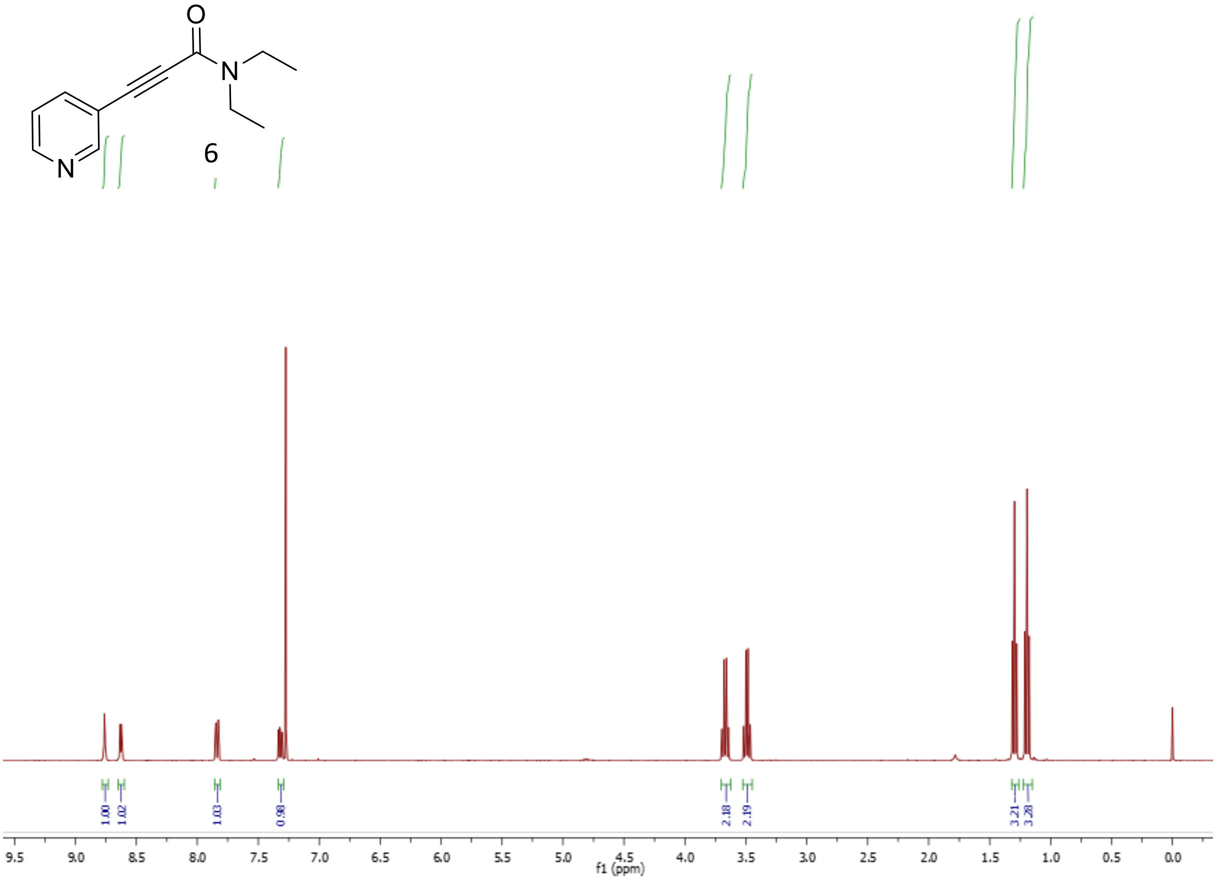
O
N
N
6
1.00
1.02
1.03
0.98
2.18
2.19
3.21
3.28
f1 (ppm)

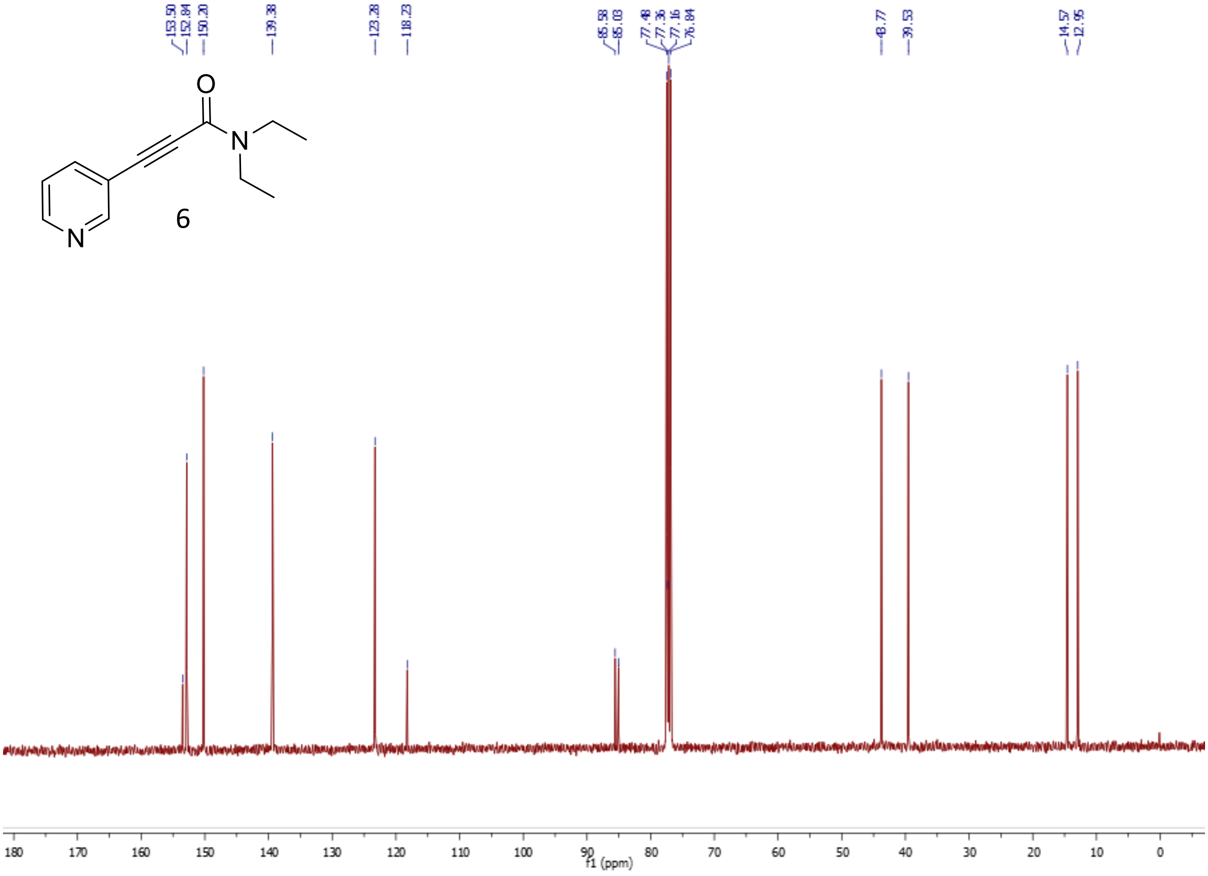
153.50
152.84
150.20
139.38
123.28
118.23
85.58
85.03
77.48
77.36
77.16
76.84
43.77
39.53
14.57
12.95
O
N
N
6
f1 (ppm)

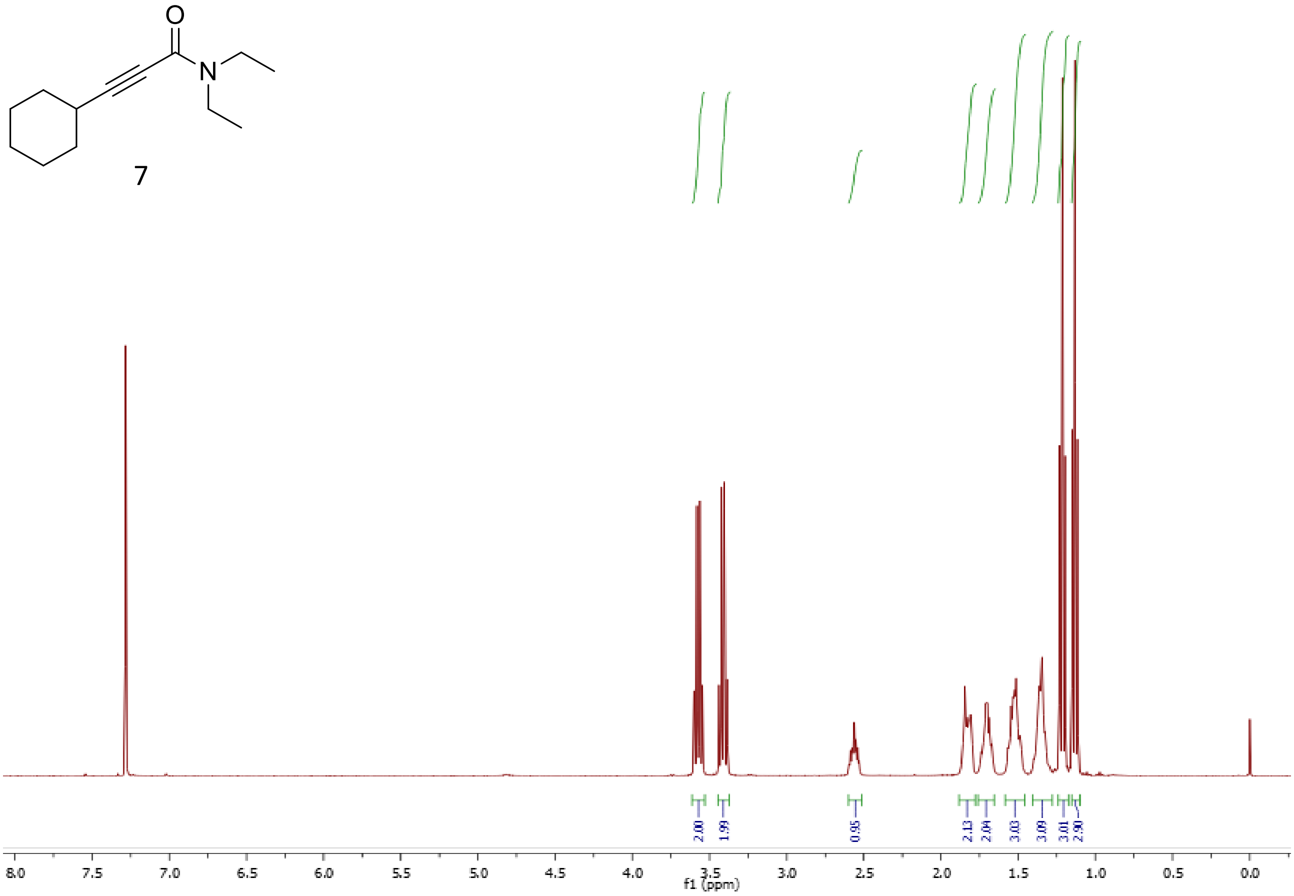

7

2.00
1.99
0.95
2.13
2.04
3.03
3.09
3.01
2.90
8.0 7.5 7.0 6.5 6.0 5.5 5.0 4.5 4.0 3.5 3.0 2.5 2.0 1.5 1.0 0.5 0.0
f1 (ppm)

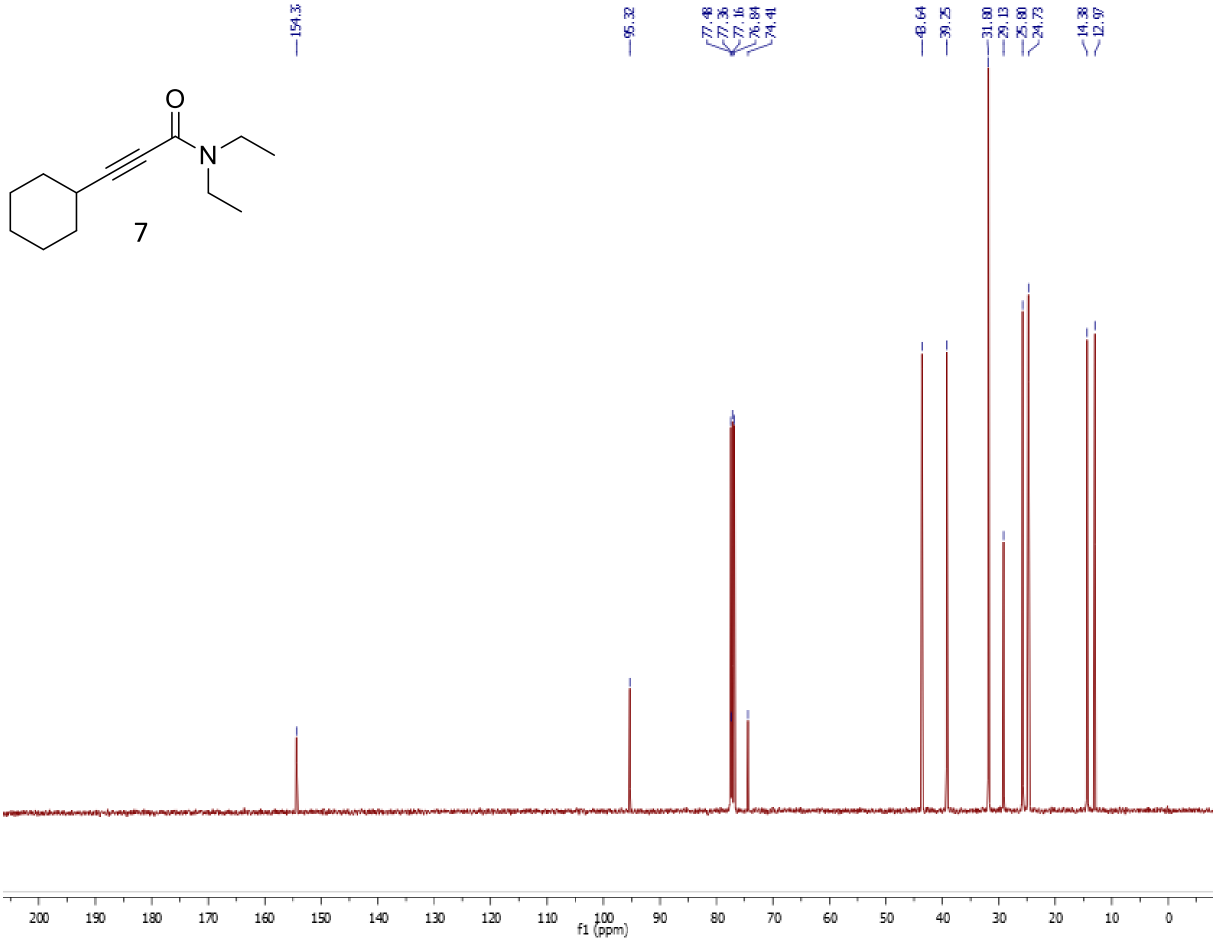

7

154.3
95.32
77.48
77.36
77.16
76.84
74.41
43.64
39.25
31.80
29.13
25.80
24.73
14.38
12.97
200 190 180 170 160 150 140 130 120 110 100 90 80 70 60 50 40 30 20 10 0
f1 (ppm)

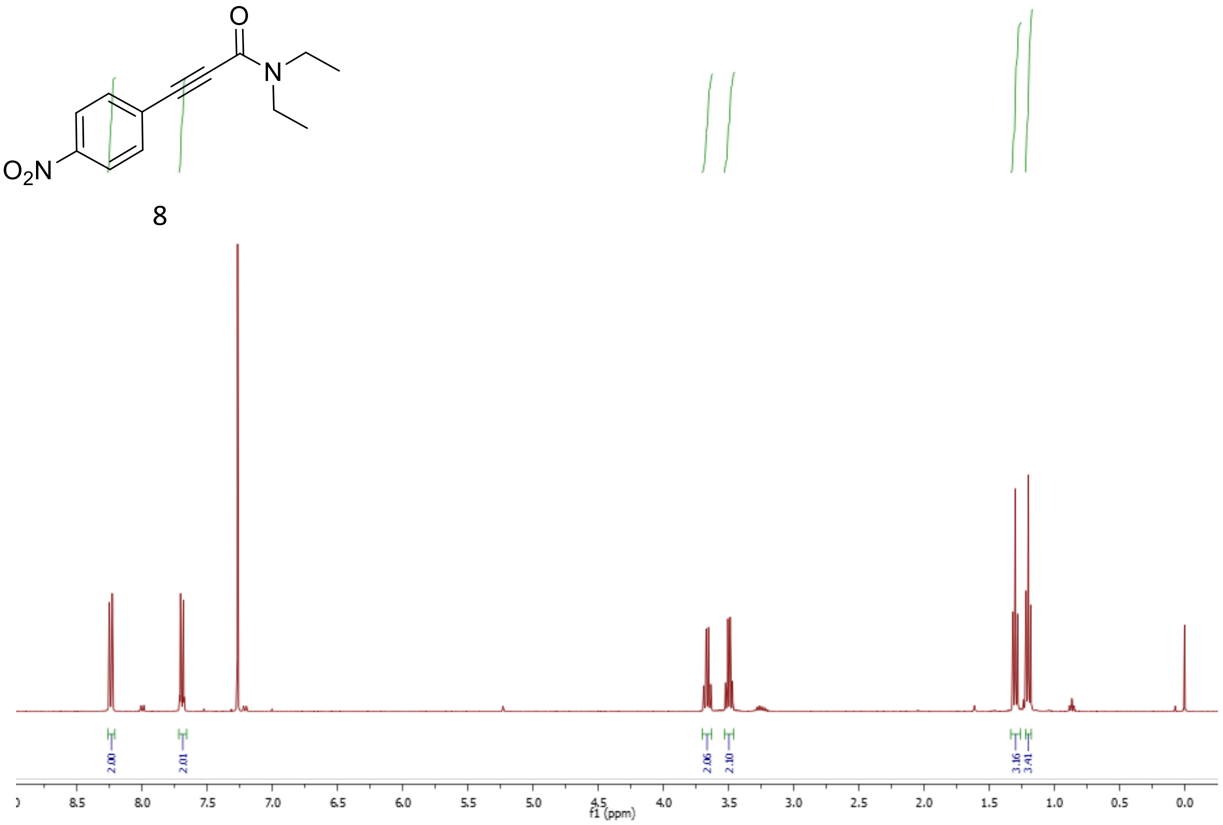

O_2N
O
N
f1 (ppm)

8

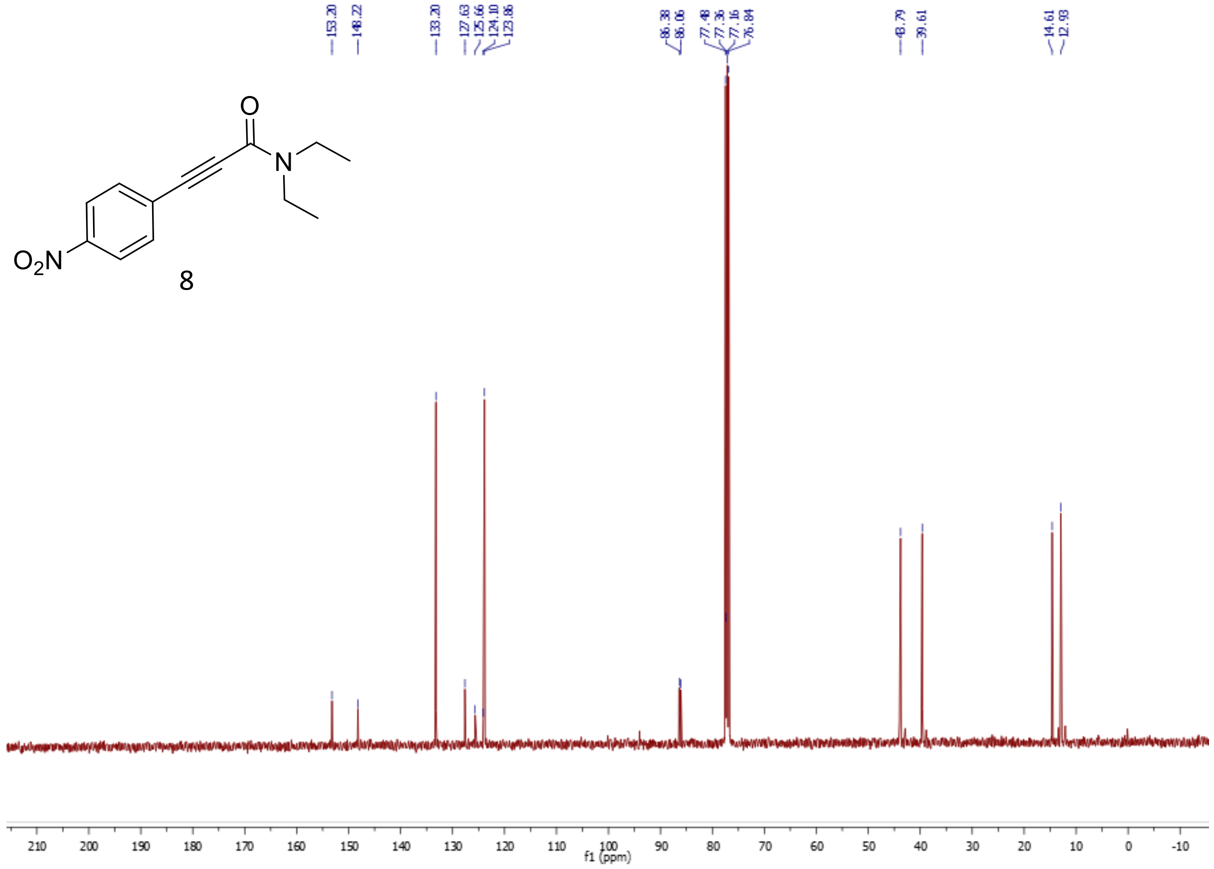

153.20
148.22
133.20
127.63
125.66
124.10
123.86
86.38
86.06
77.48
77.36
77.16
76.84
43.79
39.61
14.61
12.93
O_2N
O
N
f1 (ppm)

8

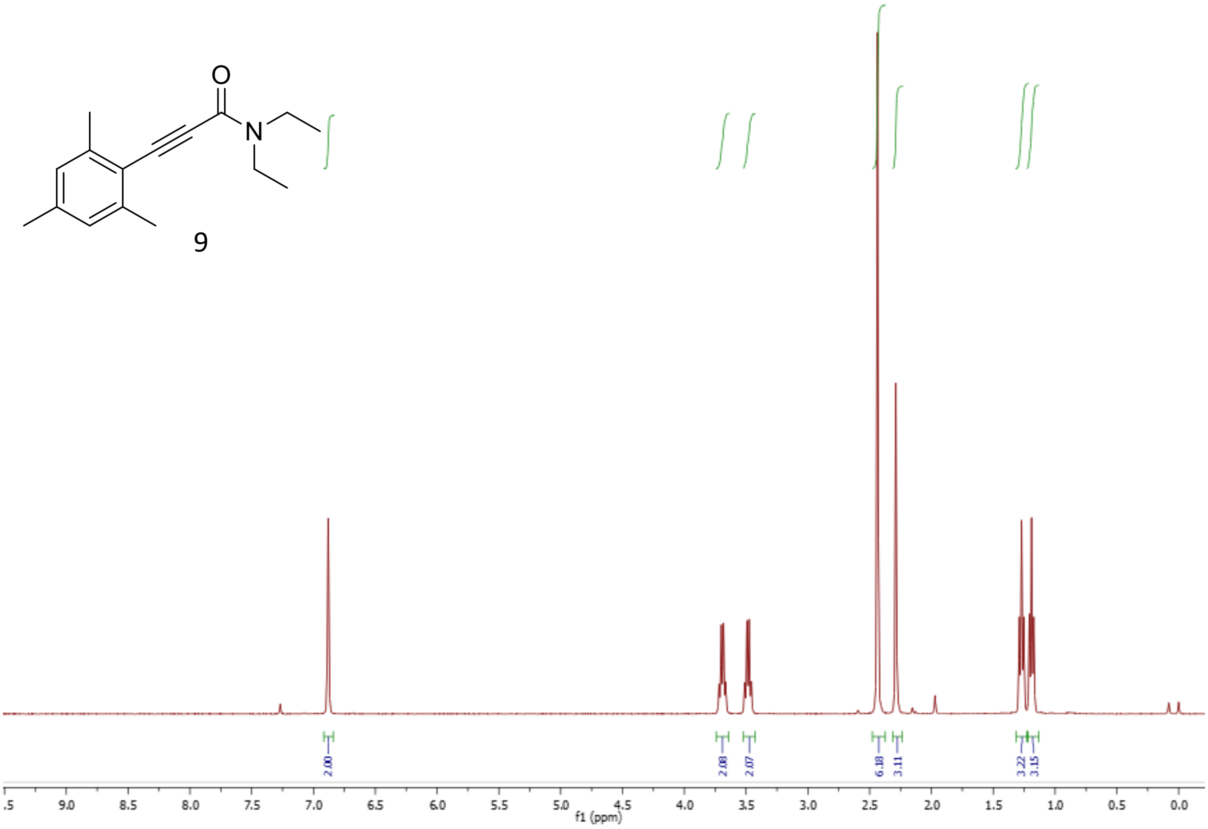
O
N
9
2.00
2.08
2.07
6.18
3.11
3.22
3.15
f1 (ppm)

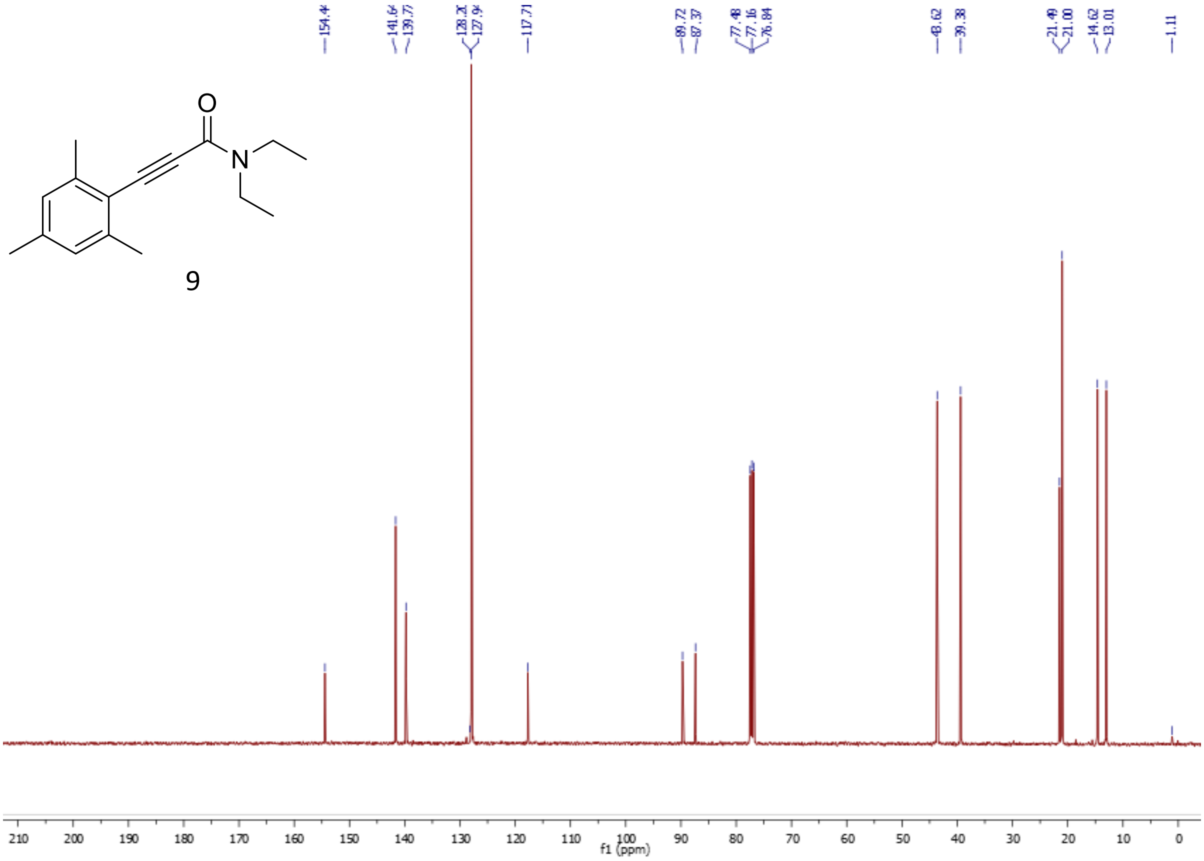
154.44
141.64
139.77
128.20
127.94
117.71
89.72
87.37
77.48
77.16
76.84
43.62
39.38
21.49
21.00
14.62
13.01
1.11
O
N
9
f1 (ppm)

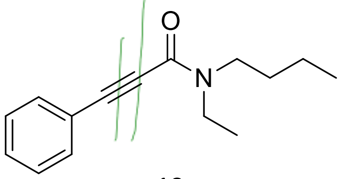

10

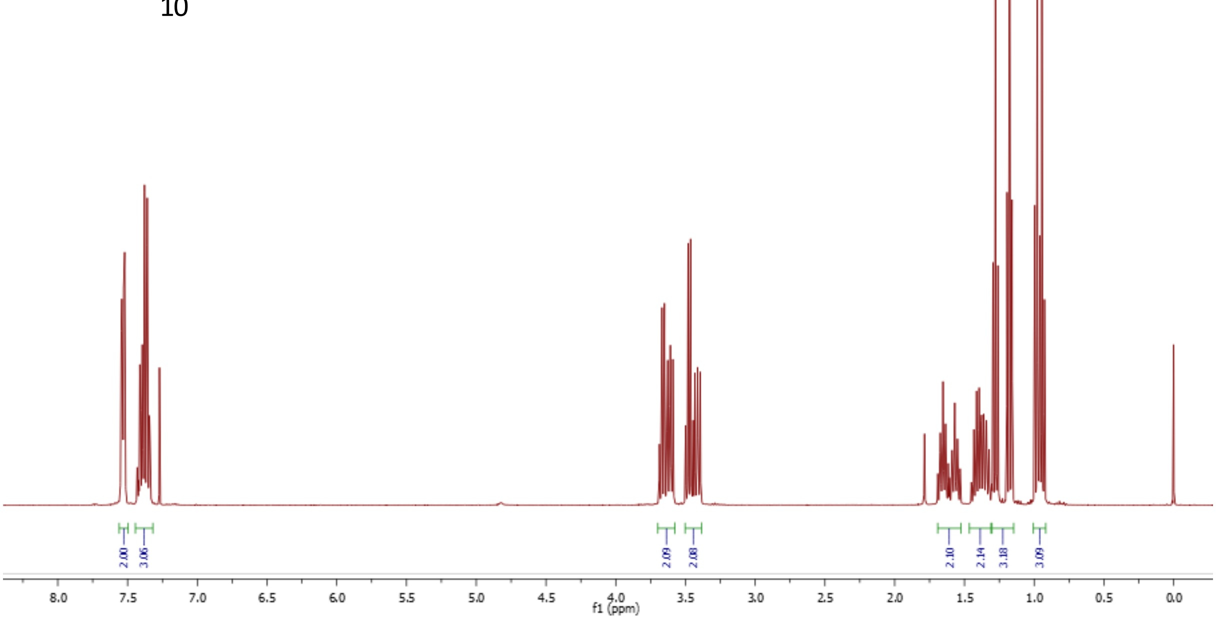

2.00
3.06
2.09
2.08
2.10
2.14
3.18
3.09
8.0
7.5
7.0
6.5
6.0
5.5
5.0
4.5
4.0
3.5
3.0
2.5
2.0
1.5
1.0
0.5
0.0
f1 (ppm)

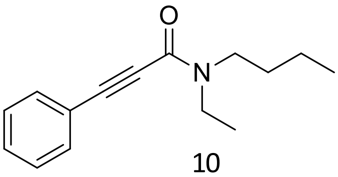

10

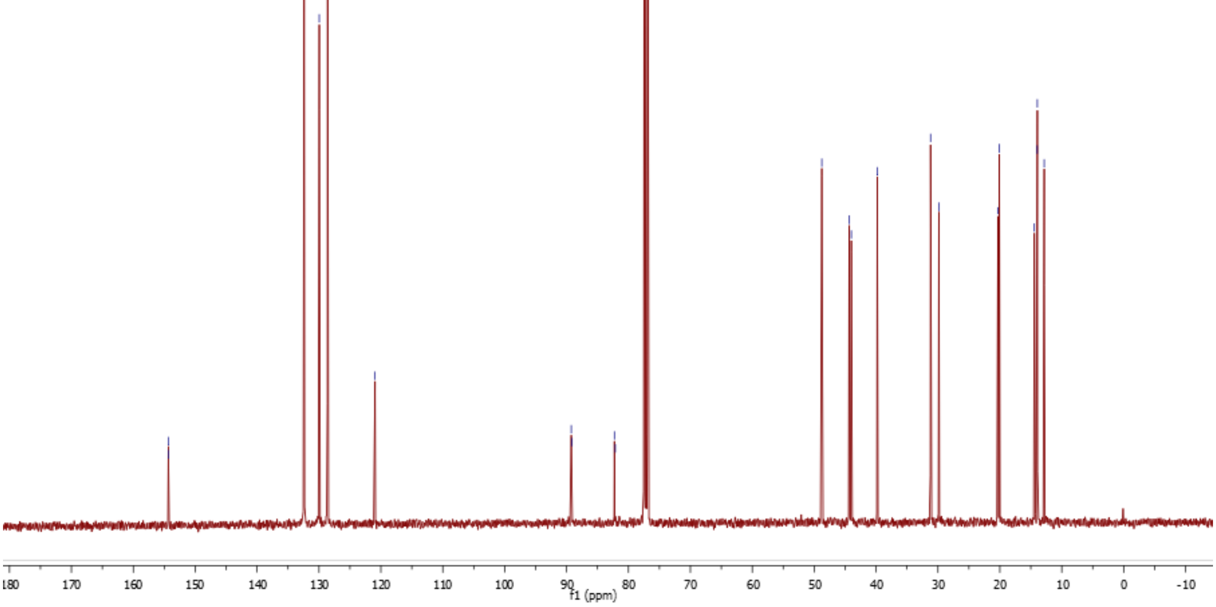

48.76
44.00
39.81
31.19
29.85
20.31
20.09
14.43
13.96
13.94
12.84
180
170
160
150
140
130
120
110
100
90
80
70
60
50
40
30
20
10
0
-10
f1 (ppm)

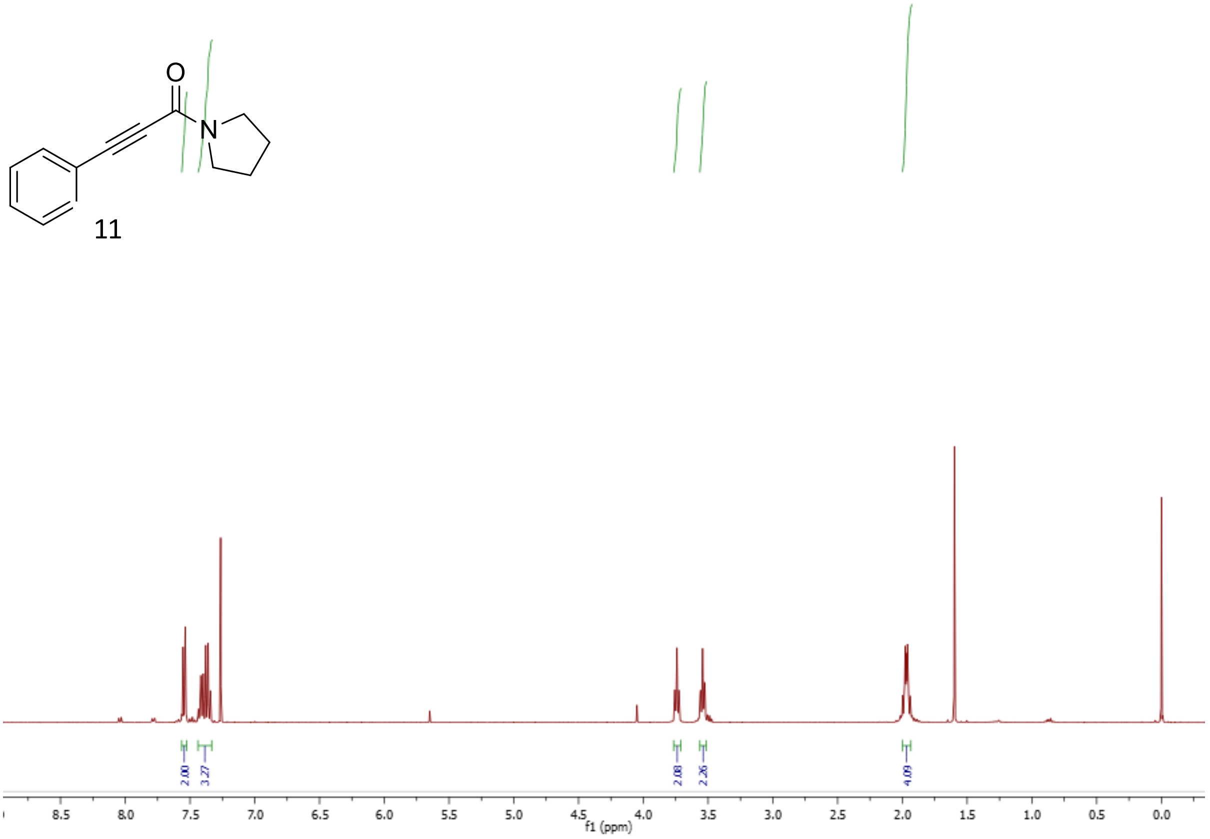
11
2.00
3.27
2.08
2.26
4.09
f1 (ppm)

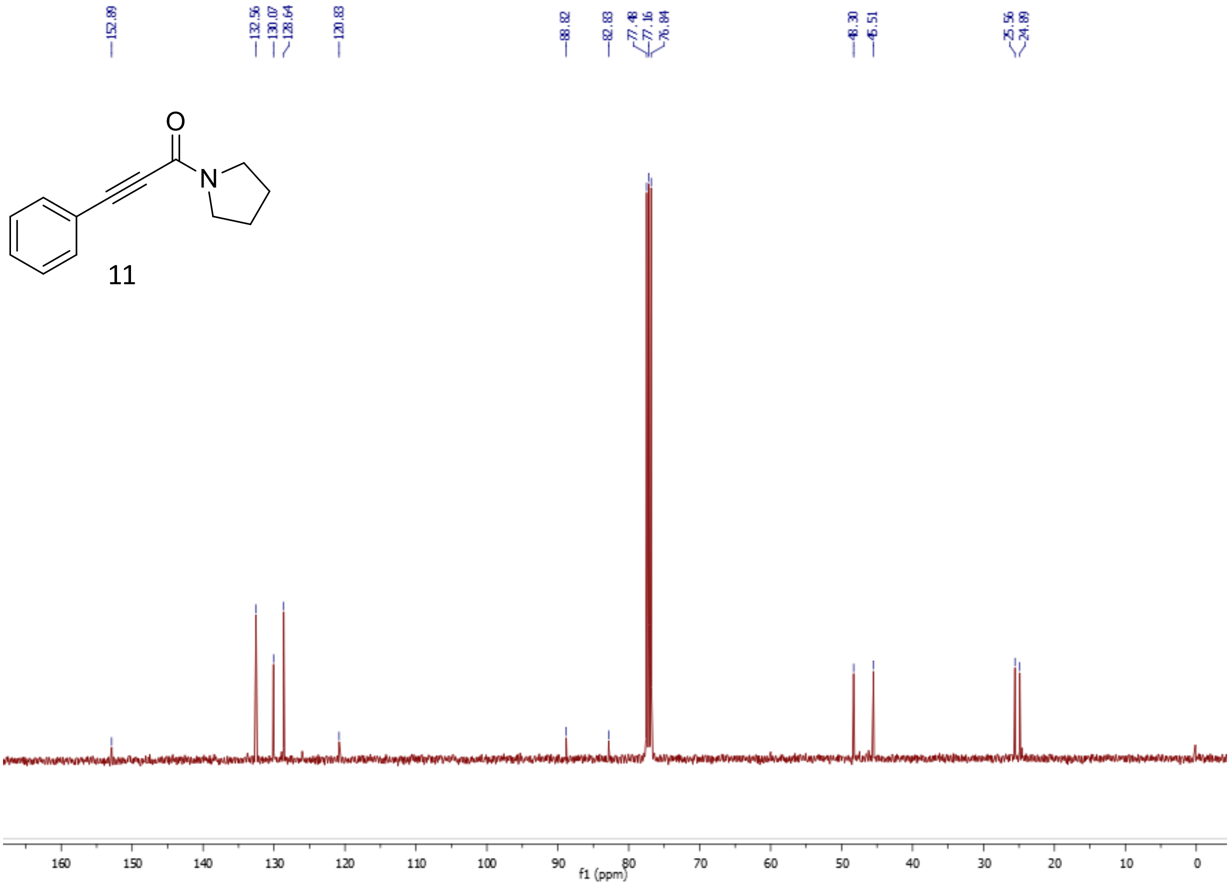
152.89
132.56
130.07
128.64
120.83
88.82
82.83
77.48
77.16
76.84
48.30
45.51
25.56
24.89
11
f1 (ppm)

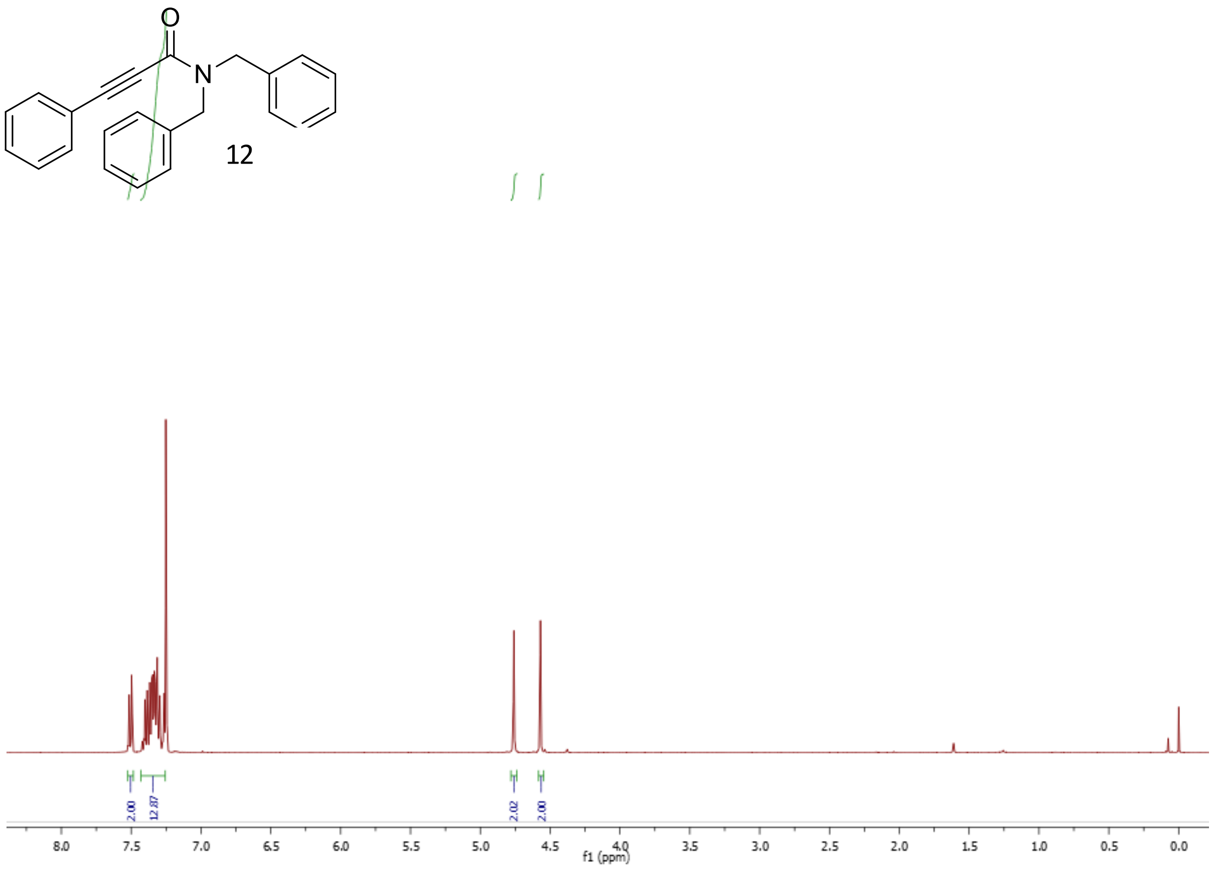
O
N
12
2.00
12.87
2.02
2.00
8.0
7.5
7.0
6.5
6.0
5.5
5.0
4.5
4.0
3.5
3.0
2.5
2.0
1.5
1.0
0.5
0.0
f1 (ppm)

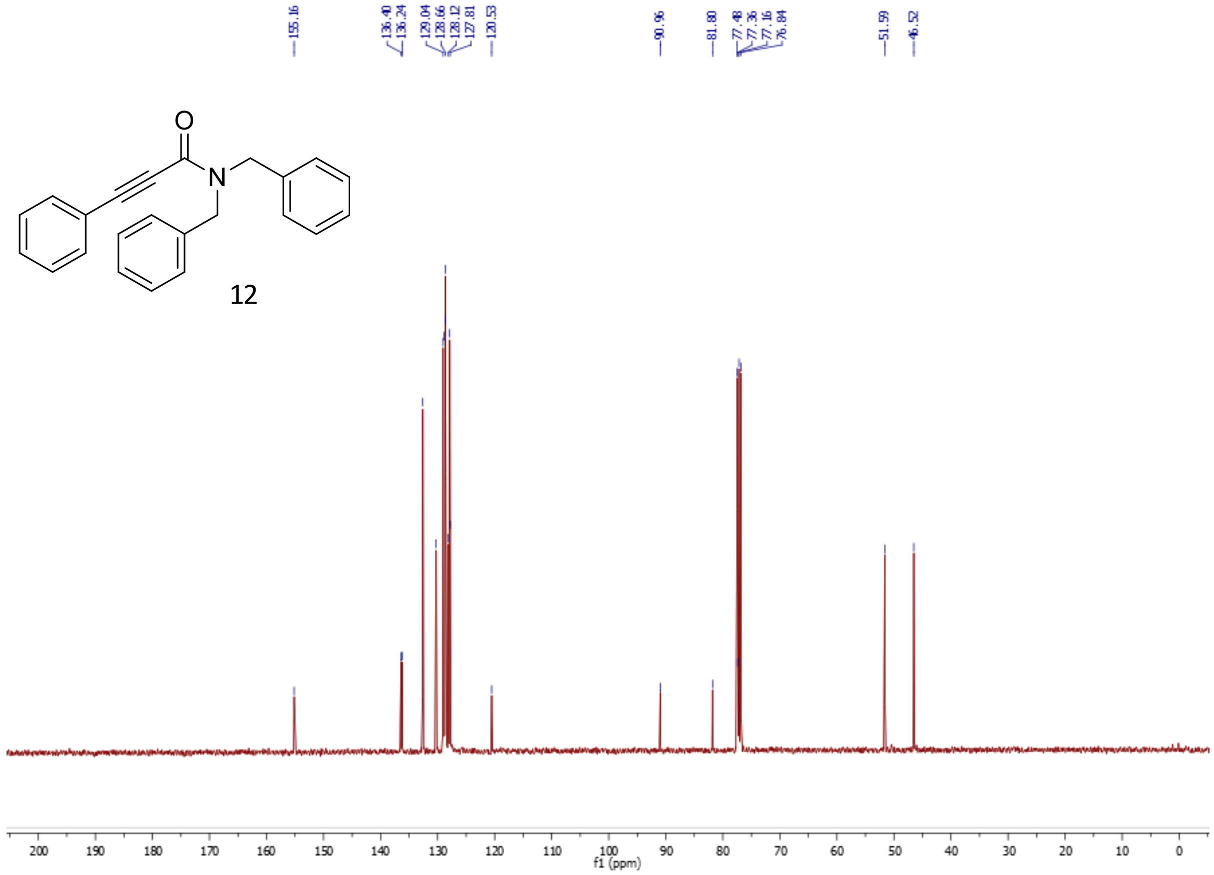
155.16
136.40
136.24
129.04
128.66
128.12
127.81
120.53
90.96
81.80
77.48
77.36
77.16
76.84
51.59
46.52
O
N
12
200
190
180
170
160
150
140
130
120
110
100
90
80
70
60
50
40
30
20
10
0
f1 (ppm)

N,*N*-dibenzyl-3-phenylpropiolamide (12)

Magnified region of ^{13}C NMR

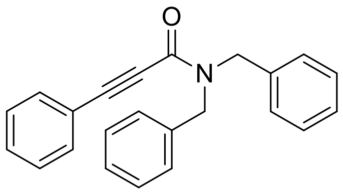

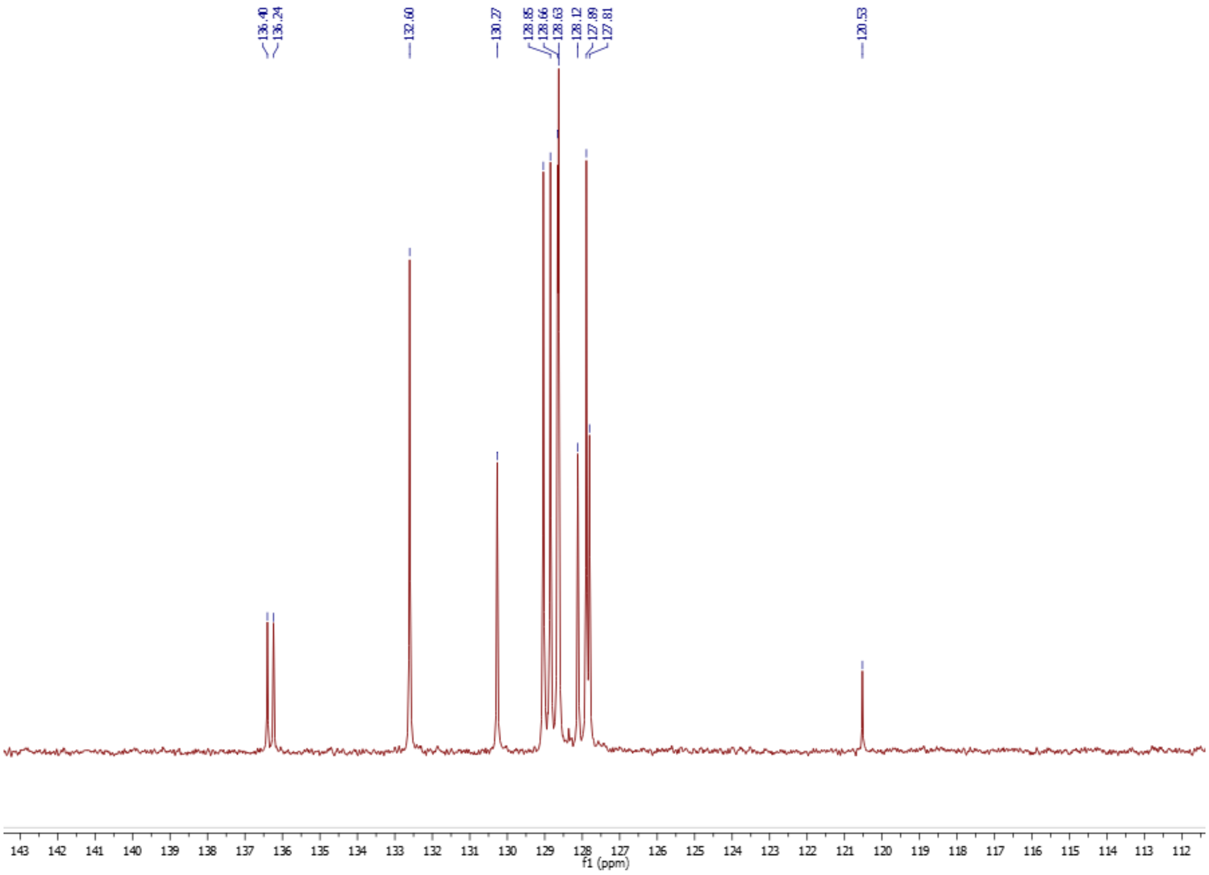

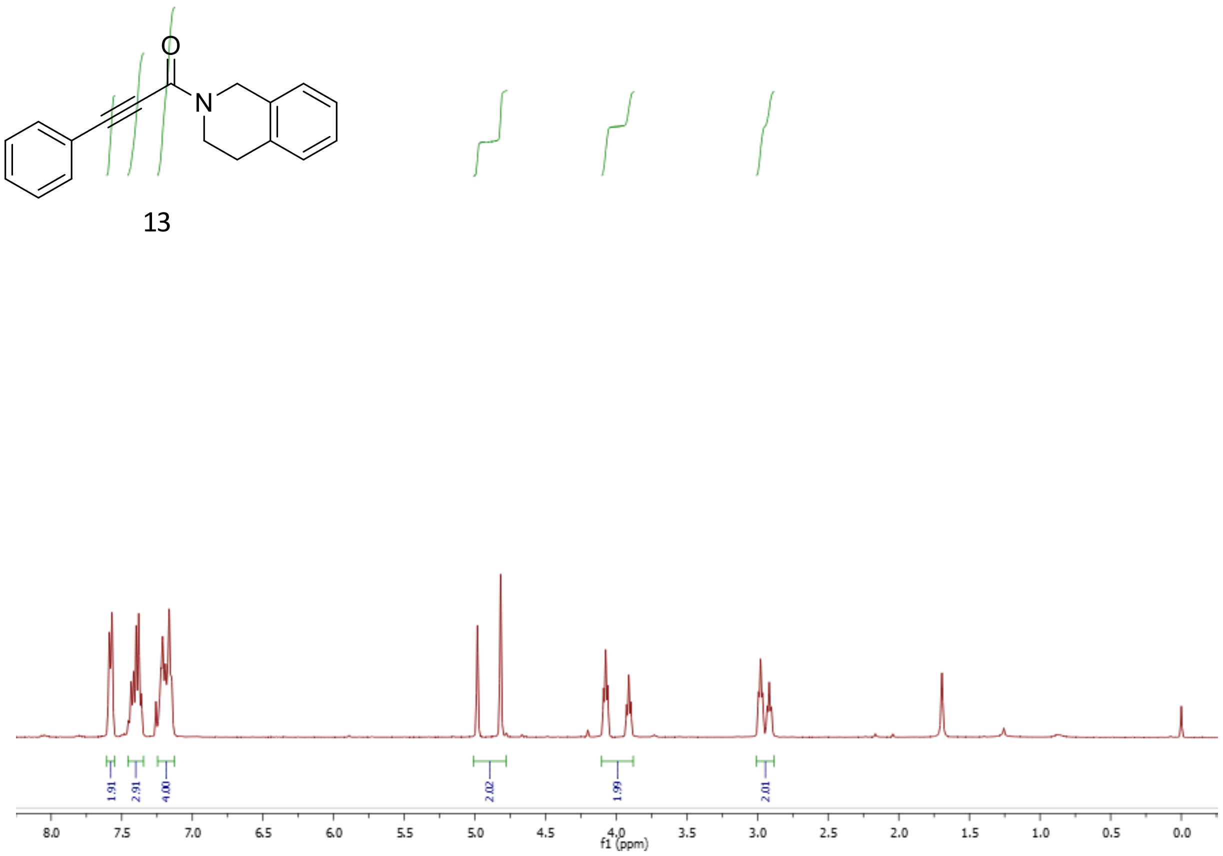

13
1.91
2.91
4.00
2.02
1.99
2.01
8.0 7.5 7.0 6.5 6.0 5.5 5.0 4.5 4.0 3.5 3.0 2.5 2.0 1.5 1.0 0.5 0.0
f1 (ppm)

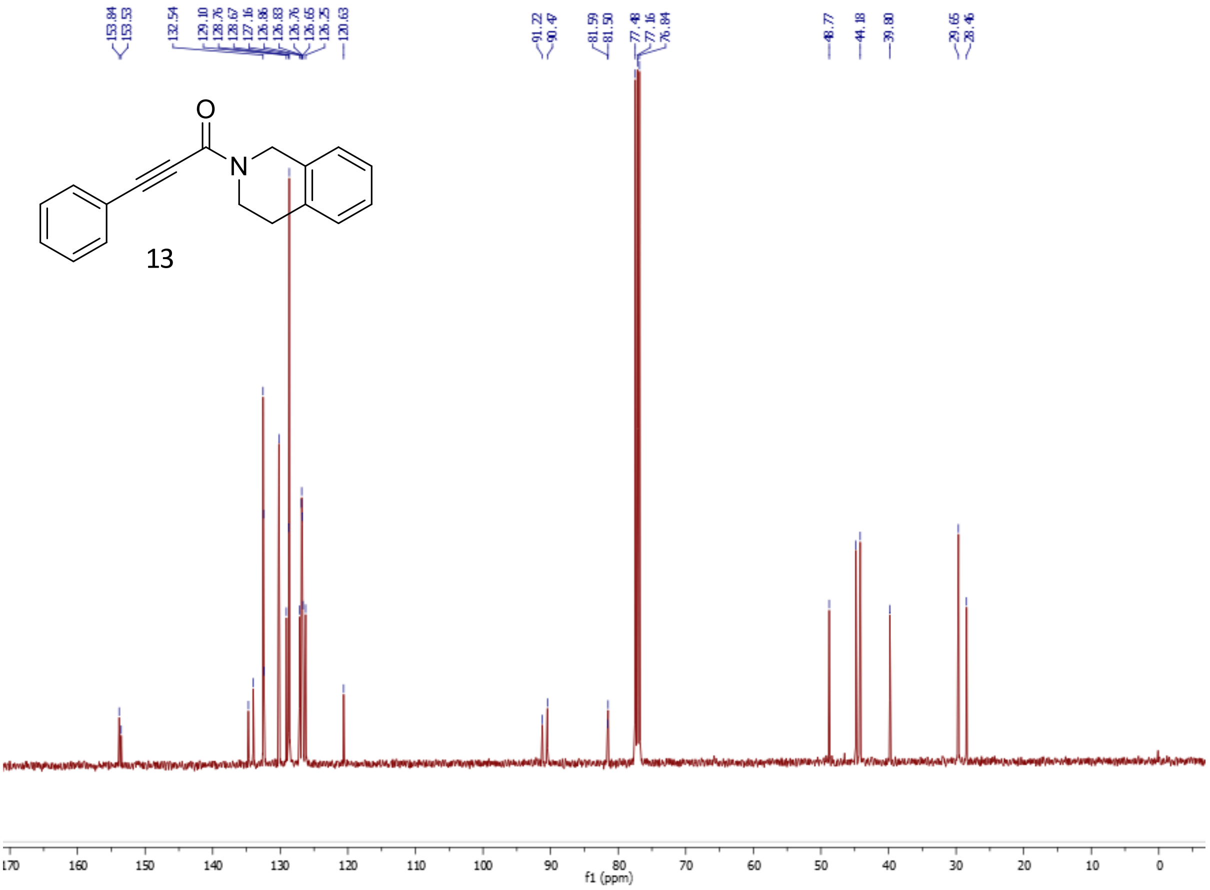

153.84
153.53
132.54
129.10
128.76
128.67
127.16
126.86
126.83
126.76
126.65
126.25
120.63
91.22
90.47
81.59
81.50
77.48
77.16
76.84
48.77
44.18
39.80
29.65
28.46
13
170 160 150 140 130 120 110 100 90 80 70 60 50 40 30 20 10 0
f1 (ppm)

1-(3,4-dihydroisoquinolin-2(1H)-yl)-3-phenylprop-2-yn-1-one (13)

Magnified region of ^{13}C NMR

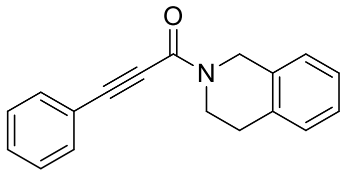

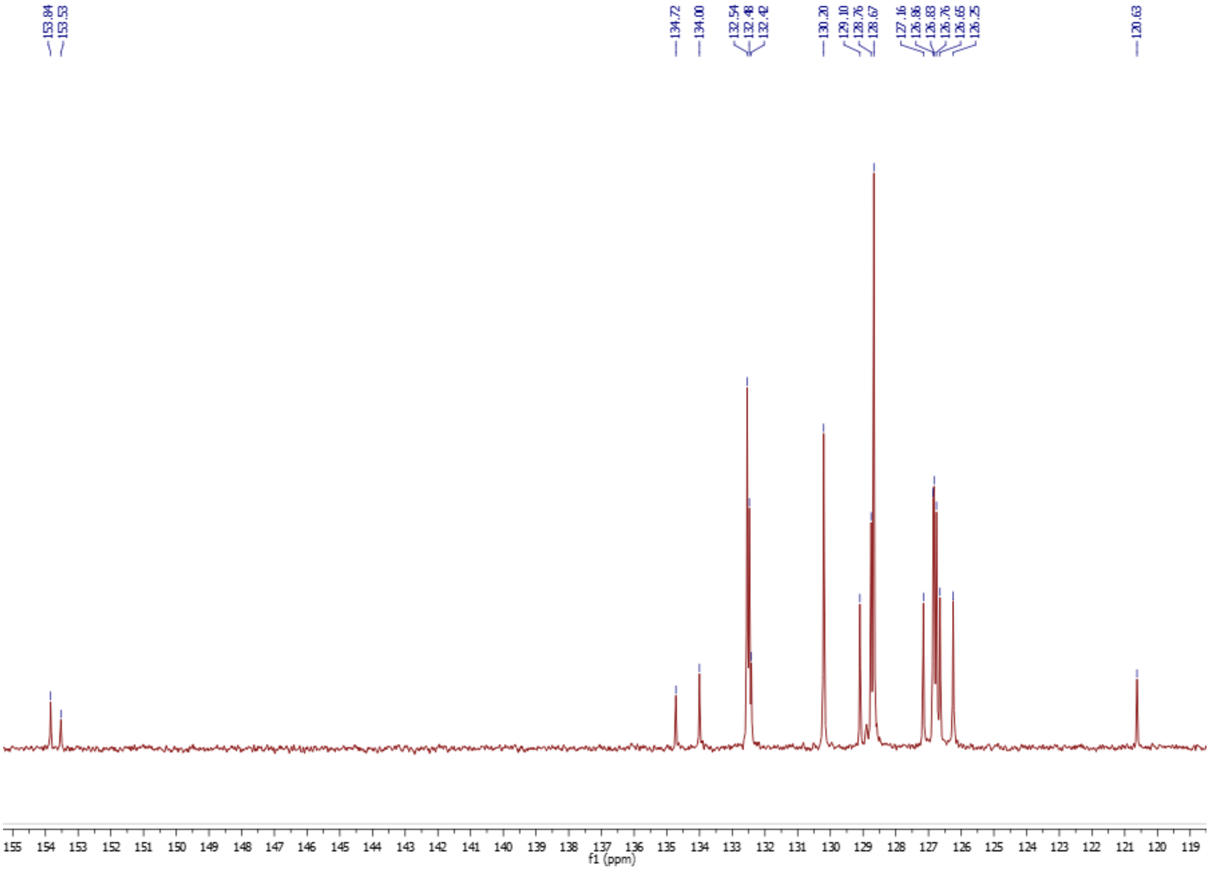

5. Example of IR spectra

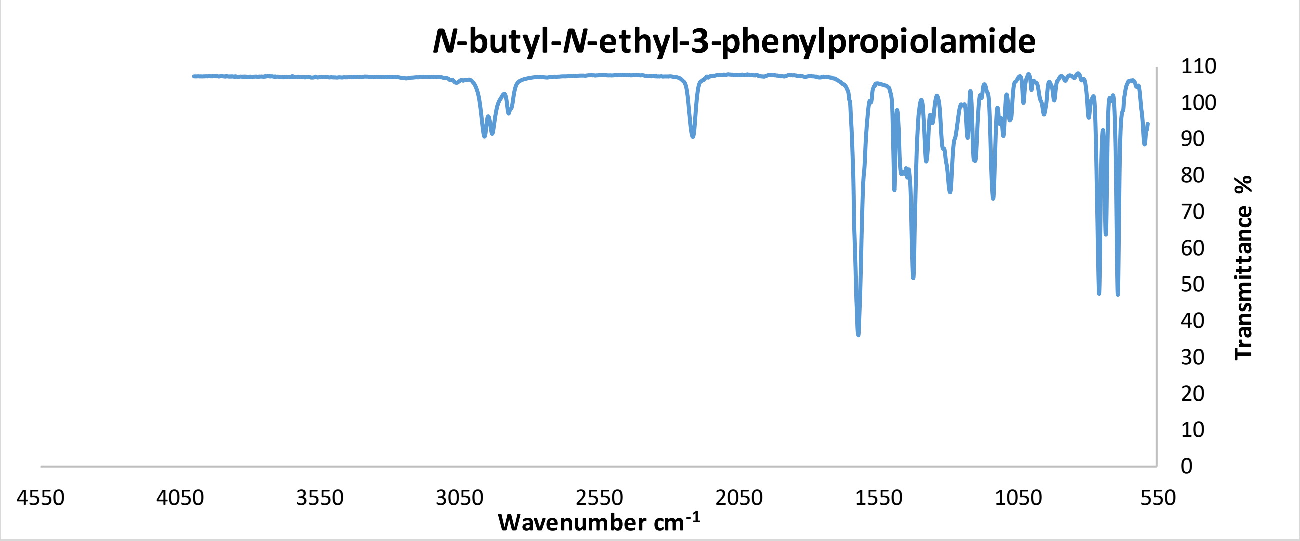

6. References

[1] J-J. Wu, Y. Li, H-Y. Zhou, A-H. Wen, C-C. Lun, S-Y. Yao, Z. Ke, and B-H. Ye, *ACS Catal*., **2016**, *6*, 1263 - 1267.

[2] B. Gabriele, G. Salerno, L. Veltri, M. Costa, *J. Organomet. Chem*., **2001**, *622*, 84 - 88.

[3] R. S. Mane and B. M. Bhanage, *J. Org. Chem.,* **2016**, *81*, 4974-4980.

[4] J. Hwang, J. Choi, K. Park, W. Kim, K. H. Song, and S. Lee, *Eur. J. Org. Chem*., **2015**, 2235 - 2243.
